# US-born and foreign-born life expectancy by race and Hispanic origin before and during the COVID-19 pandemic in the United States

**DOI:** 10.1016/j.socscimed.2025.118191

**Published:** 2025-05-13

**Authors:** Eugenio Paglino, Irma T. Elo

**Affiliations:** a Helsinki Institute for Demography and Population Health, Faculty of Social Sciences, University of Helsinki, Finland; b Max Planck – University of Helsinki Center for Social Inequalities in Population Health, Finland; c Department of Sociology and Population Studies Center, University of Pennsylvania, United States

**Keywords:** Immigration, Life expectancy, Race and ethnicity, COVID-19

## Abstract

Interdisciplinary health scholarship has long documented the lower mortality of the foreign-born compared to the US-born populations. In this study, we examine the impact of the COVID-19 pandemic on this migrant mortality advantage in 2020, 2021 and 2022 compared to 2017–2019 and estimate the contributions of the foreign-born population to US life expectancy by race and Hispanic Origin. In 2020, life expectancy declined more among all foreign-born subgroups than their US-born counterparts. The overall foreign-born life expectancy advantage declined by 1.0 year for women by 2.4 years for men and their contribution to US life expectancy declined by 0.13 years for women and 0.30 years for men. In 2021, mortality continued to increase among all US-born subgroups, but it declined among all foreign-born populations and their contributions to US life expectancy recovered their pre-pandemic size for women, but not for men. In 2022, both US-born and foreign-born life expectancies increased and the foreign-born contribution to US life expectancy exceeded its pre-pandemic size for women but not for men. At the same time, however, life expectancies for US-born and foreign-born men and women in 2022 remained below their level in 2017–2019. Among the foreign-born subgroups, Hispanic men experienced both the largest decline in life expectancy between 2017 and 2019 and 2020 and the slowest recovery by 2022. Overall, the U.S. experienced one of the largest life expectancy declines among high-income countries. This decline would have been even larger without the life expectancy gains of the foreign-born population in 2021 and 2022.

## 1. Introduction

In the United States, numerous studies have documented lower mortality and higher life expectancy among the foreign-born population than the US-born population (Hendi and Ho, 2021; Mehta et al., 2016; Singh and Hiatt, 2006; Singh and Miller, 2004; Singh and Siahpush, 2001). In 2017, the foreign-born population raised US life expectancy by 0.94 years among males and by 0.83 years among females (Hendi and Ho, 2021). Beside data artifacts (Arias et al., 2010; Palloni and Arias, 2004), explanations for the migrant mortality advantage (MMA) include positive selection on health at the time of migration, a healthy migrant effect, (Abraído-Lanza et al., 1999; Sorlie et al., 1993), cultural or social buffering effects, including health behaviors (Abraído-Lanza et al., 1999; Blue and Fenelon, 2011; Fenelon, 2013; Sorlie et al., 1993), and negative health selection at return migration, the salmon bias, (Abraído-Lanza et al., 1999; Guillot et al., 2023; Pablos-Méndez, 1994; Palloni and Arias, 2004; Turra and Elo, 2008).

Despite the long-standing evidence for lower mortality among the foreign-born population, when the COVID-19 pandemic hit in 2020, foreign-born mortality rose more than that of the US-born population within each racial and Hispanic origin group (Bacong et al., 2024; Horner et al., 2022; Paglino and Elo, 2024; Riley et al., 2021). Although COVID-19 mortality was the main explanation for this increase, cardiometabolic and respiratory diseases also contributed (Paglino and Elo, 2024). The use of the term Hispanic to reflect the way data are collected and processed by the National Vital Statistics System and the Census Bureau. The greater impact of the pandemic on the foreign-born population occurred in the context of a decline in overall US life expectancy, a 1.8-year drop between 2019 and 2020. This US life expectancy decline was driven by very large reductions in life expectancy among the American Indian/Alaskan Native (4.7 years), Hispanic (4 years), and Black (3.3 years) populations with smaller drops among Asian (2 years) and White (1.4 years) populations (NVSS, 2025). US life expectancy continued to decline in 2021 with the largest decline among the AI/AN population (1.5 years). In contrast to 2020, the 2021 decline in life expectancy among the White population (0.7 years) exceeded that of Black (0.3 years), Hispanic (0.1 years), and Asian populations (0.1 years) (NVSS, 2025). What is not known is whether the decline in overall US life expectancy reflects similar mortality trends by race and nativity and whether the contribution of the foreign-born population to US life expectancy changed over the course of the pandemic.

In this paper, we contribute to the existing literature in four key ways by: 1) documenting changes in the foreign-born vis-à-vis US-born life expectancy gap between 2017 and 2022 by race and Hispanic origin; 2) introducing a new demographic decomposition method to quantify the contribution of the foreign-born population to US life expectancy and decomposing this contribution by race and Hispanic origin, age, and cause of death, 3) documenting how the foreign-born contributions to US life expectancy by race and Hispanic origin changed over the course of the pandemic; and 4) investigating whether deaths from COVID-19, natural causes excluding COVID-19, or external causes of death were primarily responsible for the patterns we observe. The results offer new insights into the heterogeneous impact of the COVID-19 pandemic on US life expectancy by race, Hispanic origin and nativity and the impact of the COVID-19 pandemic on the foreign-born contribution to US life expectancy.

## 2. Background

### 2.1. Explanations for the migrant mortality advantage: data artifacts, health selection, and cultural and behavioral factors

A large literature has consistently found that immigrants have lower mortality than the US-born population in the United States. The main explanations for this Migrant Mortality Advantage (MMA) include data artifacts, health selection at the time of in-migration and out-migration, and health-related behaviors and cultural buffering.

Studies relying on separate data sources for death counts and population estimates are likely to suffer from the numerator/denominator bias, which occurs when individuals counted in the denominator are not counted or counted in the wrong category in the numerator, which can bias death rates. Thus, the MMA has been hypothesized to result partially from data artifacts. This issue arises because population estimates in denominators typically come from censuses or nationally representative surveys, in which both race/ethnicity and place of birth are self-reported, whereas death counts in numerators come from vital registration systems in which race/ethnicity and place of birth are reported by a funeral director who obtains this information from a third party. Studies that have examined the consistency of self-reported race/ethnicity in survey data and vital statistics have concluded that misclassification is not large enough to account for most observed racial/ethnic mortality disparities (Arias et al., 2010, 2016), although it remains severe for some population subgroups (Arias et al., 2021; Goldman et al., 2023; Park et al., 2023). While the numerator/denominator bias can be important in census and vital statistics data, the foreign-born mortality advantage is also found in studies using survey data linked with mortality records in which race/ethnicity is based on self-reports in the survey that is subsequently linked to vital statistics death records (Singh and Siahpush, 2001, 2002). In the present study, we take steps to address potential misclassification in death records and population estimates as described in the Supplementary Material.

A second set of explanations for the MMA relates to health selection. The term healthy immigrant hypothesis refers to the idea that migrants to the United States might be a particularly healthy subgroup of the population in the origin country (Abraído-Lanza et al., 1999; Sorlie et al., 1993). Direct tests of this hypothesis are challenging because they involve comparing foreign-born individuals by country of birth with non-migrants in the country of origin. Abraído-Lanza et al. (1999) noted that the foreign-born Hispanic population had lower mortality than foreign-born White individuals and that the Hispanic mortality advantage compared to the White population extended to US-born Hispanic residents and concluded that the healthy immigrant hypothesis cannot explain these patterns. Direct test investigating whether multiple health measures predicted migration from Mexico to the United States similarly found only weak support for the healthy migrant hypothesis (Diaz et al., 2018; Rubalcava et al., 2008). However, others have compared the health of the foreign-born US residents to the health of the individuals in their sending countries and found immigrants to be healthier than the population in their countries of origin (Bostean, 2013; Martinez et al., 2015; Mehta and Elo, 2012; Riosmena et al., 2017). The immigrant health advantage has also been shown to be most pronounced at working ages when most migrants arrive in the U.S. and but narrows at older ages (Boen and Hummer, 2019; Riosmena et al., 2017). In addition, the longer duration of US residence has been associated with narrowing of the foreign-born mortality and health advantage hypothesized to reflect negative acculturation, exposure to structural discrimination, and economic disadvantage (Riosmena et al., 2015).

The second hypothesized mechanism related to health selection is the so called salmon bias (Pablos-Méndez, 1994). It refers to the idea that foreign-born individuals residing in the United States might be more likely to return to their country of origin when in poor health. Because the US National Vital Statistics System does not capture deaths abroad, this process would artificially lower the mortality of the foreign-born population in the United States. The evidence for the limited role of the salmon bias in the US context is both indirect (Abraído-Lanza et al., 1999; Palloni and Arias, 2004), and direct (Diaz et al., 2016; Turra and Elo, 2008). The direct test of Turra and Elo (2008) found that while the salmon bias existed for the Hispanic population, its magnitude was not large enough to explain the MMA. Studies looking directly at whether health predicts return migration to Mexico similarly led to inconsistent results, with mixed support for the salmon bias hypothesis (Arenas et al., 2015; Diaz et al., 2016; Ullmann et al., 2011).

There is substantial evidence that the prevalence of obesity (Barrington et al., 2010; Goel et al., 2004; Mehta et al., 2015) and smoking (Blue and Fenelon, 2011; Bosdriesz et al., 2013) are lower among immigrants than among the US-born population. Lower smoking prevalence in particular, has been shown to explain a large portion of the MMA (Blue and Fenelon, 2011) and of the Mexican-American mortality advantage vis-à-vis the non-Hispanic White population (Fenelon, 2013). Beyond obesity and smoking, the evidence of better health for immigrants across other health indicators is mixed (Boen and Hummer, 2019; Choi et al., 2022; Commodore-Mensah et al., 2018; Fang et al., 2012; Singh and Hiatt, 2006; Zhang et al., 2012).

### 2.2. Mortality differentials by race/ethnicity and nativity during the COVID-19 pandemic

Previous studies have documented higher COVID-19 mortality and higher excess mortality for the Hispanic and Black populations relative to the White population, especially in 2020 (Aburto et al., 2022; Andrasfay and Goldman, 2021b; Arias and Tejada-Vera, 2023; Cronin and Evans, 2021; Elo et al., 2022; Luck et al., 2023; Sáenz and Garcia, 2021). Others have documented significantly larger mortality increases from both COVID-19 and other causes of death among the foreign-born populations, including variation by race/ethnicity compared to the US-born population (Bacong et al., 2024; Horner et al., 2022; Paglino and Elo, 2024; Riley et al., 2021). These two patterns are related. For example, the disproportionate mortality increase among the foreign-born Hispanic population explained a large portion of the decline in the Hispanic mortality advantage in 2020 (Paglino and Elo, 2024). At the same time, we know that the initial widening of the overall racial and ethnic disparities in 2020 and early 2021 was followed by a narrowing and then a return to near pre-pandemic levels in late 2021 and in 2022 (Luck et al., 2023). A shift of the pandemic from urban areas with higher proportions of racial minorities to nonmetropolitan areas with majority White populations has been proposed as an explanation for the initial widening and subsequent narrowing of the overall racial/ethnic disparities (Lundberg et al., 2023). In addition, the narrowing or disappearance of initial disparities in vaccination rates by race/-ethnicity, likely contributed (Kriss et al., 2022). Finally, given that on average the foreign-born population has lower SES than the US-born population (Semega et al., 2020; U.S. Census Bureau, 2019a), the increased importance of socioeconomic status as a predictor of health during the pandemic might have contributed to its initial greater impact on the foreign-born population, especially among the Hispanic population (Montez et al., 2024). However, no study to date has examined in detail the evolution of the mortality disparities by both race/ethnicity and nativity since 2020. In this study we fill this important gap in the literature.

## 3. Data

We use 2017–2022 National Vital Statistics System (NVSS) death records obtained from the National Center for Health Statistics (NCHS) under a data user agreement. Deaths are classified by sex, age, race (single race for White, Black, and Asian populations and a category for other and multiple race), Hispanic origin, and nativity. We include deaths of residents in the 50 states and the District of Columbia. US-born residents are individuals who were born in the 50 US states or in US territories and foreign-born residents are individuals born outside the U.S. and its territories. We use place of birth information available in the restricted NVSS data to code deaths to foreign-born and US-born individuals. Deaths to foreign-born individuals include codes for Canada, Mexico, Cuba, and all other foreign countries combined. Records with missing information on race, Hispanic origin, or nativity were allocated proportionally stratifying by age, sex, race, and Hispanic origin to foreign-born and US-born categories. We combined 2017–2022 ACS data and Census Bureau’s postcensal population estimates to construct denominators for the calculation of age-specific death rates (Ruggles et al., 2022; US Census Bureau, 2020, 2023). We further adjusted for Hispanic-origin and race misclassification on death certificates (Arias et al., 2023). Further detail on our adjustment procedures is available in the Supplementary Material. The distribution of deaths and population by year, sex, and nativity for the total US population and for the race and Hispanic-origin subgroups is reported in Table 1.

## 4. Methods

We used standard techniques to construct life tables by year, sex, race, Hispanic origin, and nativity. (Preston et al., 2001). Our estimates by race and Hispanic origin closely match US official life tables for 2017–2022 (NVSS, 2025). For comparisons between the US-Born and the foreign-born individuals we use life-expectancies at age 1 (*e*_1_). This measure is preferred to life expectancy at birth because foreign-born deaths in the age group <1 would need to occur in the US soon after the child was born abroad (Hendi and Ho, 2021). Hereafter, we will refer to life expectancy at age 1 simply as life expectancy. For additional detail on life table construction and on quantifying uncertainty see the Supplementary Material.

To estimate the contribution of foreign-born residents by age, race, Hispanic origin and cause of death to US life expectancy we extend the approach of Hendi and Ho (2021) by developing a new decomposition method. This method is an extension of the stepwise decomposition (Andreev et al., 2002) in which, going from the life expectancy of a baseline population (in our case the life expectancy of the US-born population) to the total US life expectancy, the “missing” deaths and person-years contributed by the foreign-born population by race and Hispanic origin, are added to those of the US-born population in all possible permutations. For each combination, we compute the difference between the life expectancy of the population with and without the foreign-born deaths and person years contributed by each of the racial and Hispanic-origin subgroups. We then decompose each of these differences by age and cause of death with the line-integral decomposition method (Horiuchi et al., 2008). To our knowledge, there is no previous exposition in the literature of a technique to decompose the contribution of a subpopulation (e.g. the foreign-born) to the overall national life expectancy by individual characteristics, in our case by race and Hispanic origin. More details on the decomposition are included in the Supplementary Material.

## 5. Results

### 5.1. Race and Hispanic origin composition of US- and foreign-born populations

In 2017–2022, 14.4 % of male US residents and 14.8 % of female US residents were foreign-born (Table 1). These proportions varied by race and Hispanic origin. The proportion foreign born was largest among Asian males (64.5 %) and females (69.0 %). It was also high among Hispanic males (33.9 %) and females (33.7 %), but much smaller among the Black (10.8 % of males and 10.6 % of females), White (4.6 % of males and 4.8 % of females), and multi-race (9.0 % of males and 9.5 % of females) populations. Among the entire foreign-born population, Hispanic males (44.5 %) and females (41.2 %) accounted for the largest fraction followed by Asian males (25.3 %) and females (28.0 %), White males (19.2 %) and females (19.5 %), and Black males (9.1 %) and females (9.3 %). The multi race population made up 2 % of the foreign-born population for both sexes. Among the US-born population, White males (67.9 %) and females (66.8 %) made up the largest share followed by Hispanic males (14.7 %), Hispanic females (14.1 %), Black males (12.7 %) and Black females (13.6 %). Multi-race males and females represented 3.4 % and Asian males and females less than three percent of the US-born population. The remainder of the paper focuses on Asian, Black, Hispanic-origin, and White populations, which make up 98 % of the total foreign-born population and close to 97 % of the US-born population.

### 5.2. Life expectancy by sex, race, Hispanic origin, and nativity

To address our first question, in Table 2A, we report life expectancies by nativity, with estimates disaggregated by race and Hispanic origin in Table 2B. We have included estimates of uncertainty in Table S1. In 2017–2019, the foreign-born life expectancy was 4.9 years higher for females and 5.8 years higher for males compared to their US-born counterparts. The race-specific foreign-born advantage was highest among the Black population (females: 7.2 years, males: 10.3 years), followed by the White population (females: 3.4 years, males: 4.1 years), the Hispanic population (females: 2.6 years, males: 3.7 years), and the Asian population (females: 0.2 years, males: 1.0 years).

In 2020, life expectancy declined for both foreign-born and US-born residents, but this decline was greater for the foreign-born population. The life expectancy disparity between the foreign-born and US-born populations declined by 1.0 year for females and by 2.4 years for males (Table 2A). Among men, the foreign-born advantage declined the most for Hispanic men (3.2 years), followed by Black men (2.3 years), and White men (1 year). Foreign-born Asian men had higher life expectancy than US-born Asian men in 2017–2019, but lower life expectancy in 2020. Among women, the largest reduction in the foreign-born advantage in 2020 compared to 2017–2019 was among Black women (1.7 years), followed by Hispanic women (1.2 years), and White women (0.3 years). Among Asian women the small foreign-born advantage (0.2 years) turned to a 2.0-year disadvantage (Table 2B).

In 2021, life expectancies increased among both foreign-born men and women among all racial/ethnic subgroups. In contrast, life expectancy continued to decline among all US-born subgroups (Table 2B). Thus in 2021, the foreign-born population recovered some of its advantage relative to the US-born population. The overall foreign-born advantage increased to 4.8 years for females and to 5.1 years for males, but both remained below their pre-pandemic advantages of 4.9 years and 5.8 years, respectively (Table 2A). Among the racial and Hispanic-origin subgroups, Black and White foreign-born residents fully recovered their life expectancy advantage relative to their US-born counterparts due to the continued increase in mortality among the US-born populations. In contrast, the foreign-born advantage in life expectancy remained below its pre-pandemic advantage among Hispanic and Asian men and women (Table 2B).

In 2022, life expectancies increased among all foreign-born and US-born subgroups. Among the entire US population, the foreign-born advantage was 4.9 years among women, close to what it had been in 2017–2019 (4.90 years), and 5.4 years among men remaining below its pre-pandemic level of 5.8 years (Table 2A). The foreign-born life expectancy advantages by race and Hispanic origin were also below their pre-pandemic levels for most subgroups, except for Black and White men. Among Hispanic men, the foreign-born advantage remained 1.46 years below its pre-pandemic level, and among Hispanic it was 0.31 years below its pre-pandemic level. Similar patterns were observed for White women and Black women. Among Asian men and women, the pre-pandemic advantage had turned to a disadvantage by 2022 (Table 2B).

### 5.3. Foreign-born contributions to US life expectancy by sex, race, Hispanic-origin and cause of death

**Fig. 1.**
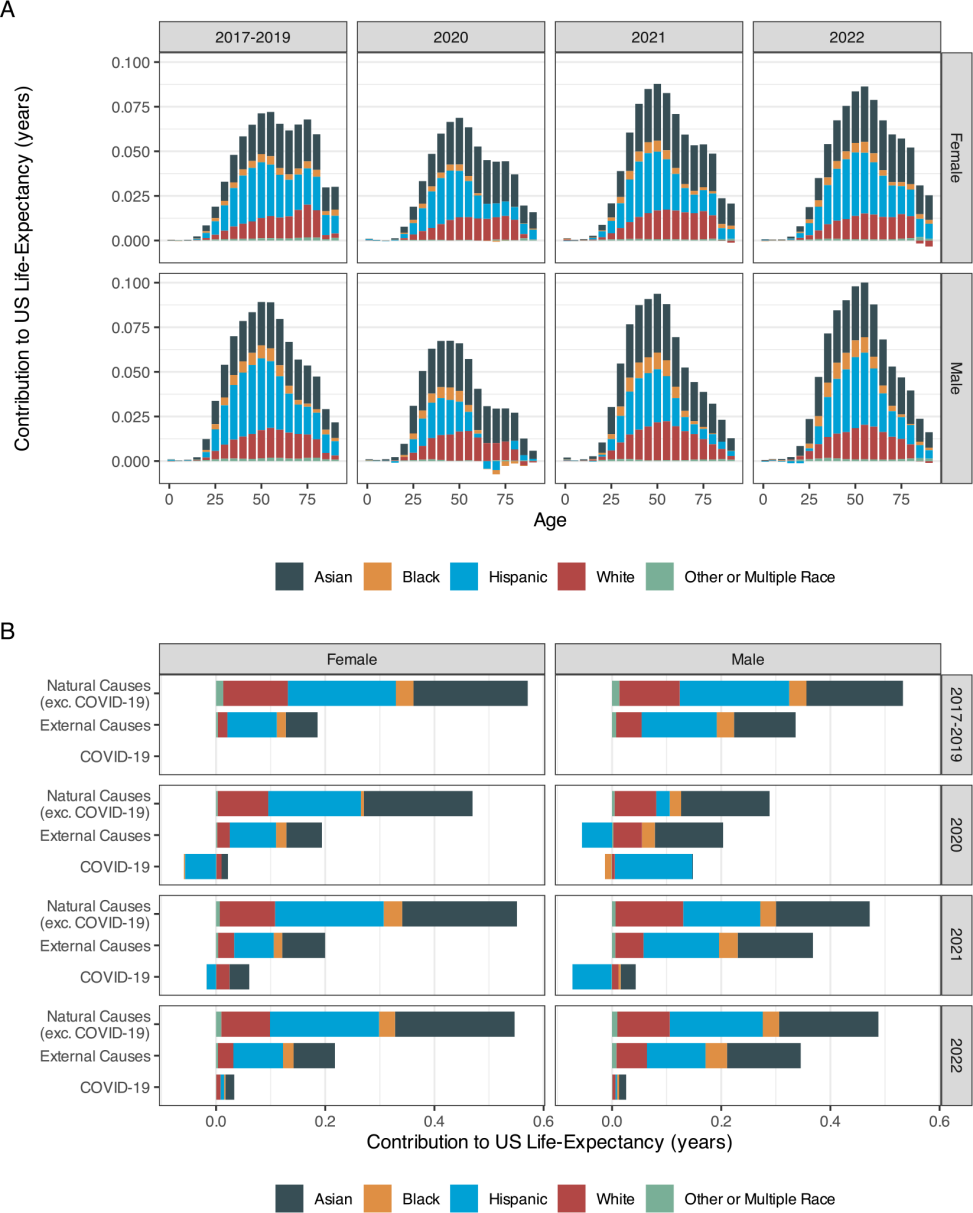
Contribution of Foreign-Born Residents to US Life Expectancy by Race, Hispanic Origin, Sex, Year, and Age (Panel A) and by Cause of Death (Panel B). Notes: Panel A presents the age-group contributions as stacked bars so that the area in each graph equals to the contributions of foreign-born residents by race and Hispanic-origin and each vertical section equals the total contribution of all foreign-born residents in the corresponding age range. Panel B presents the cause-specific contributions to life expectancy, which each color representing the contribution of the race and Hispanic origin groups.

To address our second question, we estimate the contributions of foreign-born subgroups by race and Hispanic origin to sex-specific overall US life expectancies. In 2017–2019, foreign-born females extended US female life expectancy by 0.76 years and foreign-born males extended US male life expectancy by 0.87 years (Table 3A). Of the total foreign-born positive contributions, the Hispanic share was the largest among both females (0.29 years, 38 %) and males (0.34 years, 39 %). The contributions of Asian females (0.27 years, 35 %) and males (0.29 years, 33 %) were also sizable, followed by White, Black and multiple or other race populations, reflecting the subgroups’ level of mortality and their population size (Fig. 1, Table S2, and Fig. S1). Mortality at working ages (25–64) made the largest contribution, accounting for 57 % of the female and 66 % of the male foreign-born contributions to total US life expectancy (Table 3A). This age pattern holds for all racial and Hispanic-origin subgroups (Table S3).

In 2020, the size of the foreign-born contribution to US life expectancy was reduced and this decline was particularly large for the Hispanic population, declining by 31 % for females and by 67 % for males (Table S2). As a result, the contribution of the foreign-born Asian population now exceeded that of the Hispanic population, accounting for 44 % of the overall foreign-born contribution to female life expectancy and 50 % to male life expectancy. In 2021, due to the declining foreign-born mortality and the continued increase in US-born mortality, the size of foreign-born contributions to the US life expectancy rebounded, especially for females (Table 3A). Among the foreign-born population, Asian men and women continued to make the largest contribution to US life expectancy in 2021. Even with declines in US-born mortality in 2022, the overall foreign-born contribution continued to increase, particularly among males. Asian males and females continued to make the largest positive contribution to US life expectancy in 2022 (Fig. 1A and Table S2). This reflects both their population size and their low mortality (Fig. S1).

Table 3B presents foreign-born cause-specific contributions to US life expectancy. These contributions by race and Hispanic origin are shown in Fig. 1B and in Table S4. Lower foreign-born mortality from natural and external causes explained why the foreign-born population extended US life expectancy in 2017–2019. The decline in the foreign-born contribution to US life expectancy in 2020 among women was driven both by COVID-19, which was responsible for 38 % of the decline in this contribution, and by declines in the contribution of natural causes (69 %), with a small but positive increase in the contribution of external causes (7 % of the change). Among males, COVID-19 explains 40 % of the decline in the contribution, with a decline in the contribution of both natural (33 %) and external causes (27 %) explaining the residual change (Table 3B). In 2021, the contributions of natural causes approached their pre-pandemic levels, and COVID-19 reduced the foreign-born life expectancy advantage only among Hispanic men and women. External-cause mortality was higher among the US-born than the foreign-born populations throughout the period and this US-born disadvantage increased somewhat in 2021, especially among males (Table 3B and Fig. 1B). In 2022, the contribution of natural and external causes to the foreign-born advantage returned to the pre-pandemic pattern and the foreign-born disadvantage in COVID-19 disappeared among all subgroups (Table 3B and Fig. 1B). See Fig. S2 for results by detailed natural causes.

## 6. Discussion

In this paper, we document the differential trends in US mortality and life expectancy by race, Hispanic-origin and nativity and examine the contributions of foreign-born populations to US life expectancy between 2017 and 2019 and 2022. We find that in 2020, the COVID-19 pandemic increased mortality among both US-born and foreign-born populations, but it disproportionately impacted foreign-born US residents, thus reducing the US-born versus foreign-born mortality disparities. These findings confirm earlier studies documenting a more severe impact of the pandemic on the foreign-born population in Minnesota (Horner et al., 2022), California (Riley et al., 2021), and nationally (Bacong et al., 2024; Paglino and Elo, 2024) in 2020. We also document larger life expectancy declines for the Black and Hispanic populations relative to the White population, confirming earlier findings (Andrasfay & Goldman, 2021a, 2021b). In contrast with the patterns observed in 2020, in 2021, when mortality continued to increase among the US-born population, it declined among all foreign-born subgroups. In 2022, mortality fell among both foreign-born and US-born residents, but the pace of the reduction was faster among several foreign-born subgroups. Life expectancies increased across all population subgroups by race and Hispanic origin with the gains being larger for the Black and the Hispanic population relative to the White population. However, it is important to note that by 2022, no population subgroup, except US-born Asian men and women, had recovered its pre-pandemic life expectancy. In addition, the foreign-born mortality advantage remained below its 2017–2019 level for all groups except among Black and White men.

Before the COVID-19 pandemic the foreign-born population contributed 0.76 years to female life expectancy and 0.87 to male life expectancy. These contributions declined markedly in 2020 but began to recover in 2021 and by 2022 exceeded pre-pandemic levels for females (0.80 years) and came close for males (0.86 years). However, mortality trends among the foreign-born subgroups varied over time such that by 2022 the foreign-born Asian contribution to US life expectancy exceeded those of the foreign-born Hispanic population that had contributed the most years prior to the pandemic in 2017–2019.

In 2020, deaths from COVID-19 alone accounted for close to 40 % of the decline in the foreign-born contribution to US life expectancy due to the higher mortality among the foreign-born than US-born residents from COVID-19 (Bacong et al., 2024; Horner et al., 2022; Paglino and Elo, 2024). The advantage that the foreign-born populations had with respect to natural cause and external cause mortality also declined in 2020 compared to 2017–2019. It is important to note that deaths from COVID-19 alone have been shown to imperfectly capture the impact of the pandemic on US mortality (Luck et al., 2023; Paglino et al., 2024) and it is likely that at least some of the reduction in the foreign-born advantage in natural cause mortality reflects COVID-19 mortality missed in official statistics.

Explanations for the foreign-born mortality advantage reviewed previously are unlikely to explain the sudden uptick in mortality among foreign-born US residents from 2017 to 2019 to 2020. For example, although health-related selection is likely to have played a role in the pre-pandemic foreign-born mortality advantage, we do not believe that changes in migration patterns induced by the pandemic could explain the patterns we observe. For example, if negative health selection of return migrants took place during the pandemic, in its absence we would have seen an even larger increase in foreign-born mortality in the first year of the pandemic. It is possible that changes in health-related characteristics of new immigrants and return migrants between 2019 and 2020 and subsequently in 2021 and 2022 could have affected foreign-born mortality. However, given that the stock of migrants increased by only 0.7 % between 2019 and 2021, and by 2.0 % between 2021 and 2022 (Migration Policy Institute, 2024; U.S. Census Bureau, 2019b) it is unlikely that changes in the composition of the foreign-born population explain the observed patters. It is possible that data artifacts could account for some fraction of the foreign-born mortality advantage, which would affect all estimates of foreign-born mortality throughout the period from 2017 to 2022, but data artifacts are unlikely to explain year-to-year changes.

The disproportionate increase in mortality among the foreign-born population is likely a combination of the highly infectious nature of COVID-19, a greater exposure of the foreign-born population to the virus when it first appeared and the impact of the pandemic on the economy. For example, there are indications that socioeconomic status became a stronger predictor of health outcomes during the pandemic, which could help explain the disproportionate mortality increase among the foreign-born population (Montez et al., 2024). In 2019, the median household income was 7 % lower among all foreign-born than US-born residents (Semega et al., 2020) and the proportion of the foreign-born population without a high-school degree was more than three times higher than among the US-born population (26 % vis-à-vis 8 %) (U.S. Census Bureau, 2019a). In addition, a high proportion of foreign-born residents work in essential occupations (Chishti and Gelatt, 2022; Riley et al., 2021) and reside in intergenerational households (Cohn et al., 2022), factors that can facilitate the spread of the virus. More limited access to healthcare by the foreign-born than the US-born population may have also made COVID-19 more deadly among foreign-born individuals (Bustamante et al., 2019; Ku, 2009). Other factors such as lower vaccination rates among racial/ethnic minority populations in the initial phase of the pandemic may have also contributed to the higher mortality among the foreign-born than the US-born population (Kriss et al., 2022).

Among the foreign-born subgroups, Hispanic males and females experienced the largest life expectancy declines in 2020 as well as the most incomplete recovery. There are several potential explanations for the greatest impact of the pandemic on the foreign-born Hispanic population. For example, the foreign-born Hispanic population is least likely to have health insurance coverage (Fuentes et al., 2022). In addition, undocumented individuals make up a larger fraction of the Hispanic foreign-born population than of the Asian or Black foreign-born residents (Budiman, 2021; Passel and Krogstad, 2023) and they may have been particularly reluctant to seek health care, especially during the first year of the pandemic due to increased immigration enforcement activities (Clark et al., 2020). Others have further shown that foreign-born Hispanic residents were particularly likely to be essential workers who were hard hit by the pandemic (Chishti and Gelatt, 2022; Riley et al., 2021) and experience crowded living conditions (Cohn et al., 2022; Hall et al., 2019). Compared to the foreign-born Black and Asian populations, the foreign-born Hispanic population is also socioeconomically more disadvantaged. The percentage of the foreign-born Hispanic population with less than high school education is 42.5 % compared to 13.7 % among foreign-born Black, 14.0 % among foreign-born Asian, and 9.8 % among foreign-born White populations, (calculations by the authors based on 2019 ACS data).

We speculate that the evolving geography of the COVID-19 pandemic from 2020 to 2022 likely played a role in the divergent mortality trends between US-born and foreign-born populations. A high fraction of foreign-born residents was concentrated in metropolitan areas, especially in central cities, with only 20 metropolitan areas being home to 64 % of the foreign-born population prior to the pandemic (Budiman, 2020) where highest COVID-19 and excess mortality were recorded in 2020. In contrast, less than 5 % of the foreign-born population lived in nonmetropolitan areas (Table S5) where the lowest COVID-19 and excess mortality were observed in 2020 (Lundberg et al., 2023; Paglino et al., 2023). In addition, foreign-born residents were overrepresented compared with US-born residents (35.0 % versus 31.7 %) across the three Census divisions with the highest relative excess mortality in 2020 (West South Central, Middle Atlantic, and Mountain divisions) and underrepresented (15.7 % versus 27.5 %) across New England and West North Central and East North Central division with the lowest relative excess mortality in 2020 (Table S6). These geographic patterns of the COVID-19 pandemic reversed in 2021 and continued to 2022 with excess mortality increasingly being concentrated in nonmetropolitan areas but declining in large metropolitan areas (Paglino et al., 2023). Further research into the latter phase of the pandemic is needed to elucidate the factors that could explain the divergent trends among the foreign-born and US-born populations and by race and Hispanic origin.

### 6.1. Limitations

This paper comes with limitations. First is the potential mismatch between the race and Hispanic-origin identification and place of birth on death certificates and in Census population estimates. We have addressed this concern by incorporating adjustment factors based on linked data that are also used in the construction of official US life tables (Arias et al., 2023). However, these correction factors are available only for the entire US population and thus it is possible that there is some variation in the reporting accuracy by nativity. Second, inaccuracies in the reporting of nativity on death certificate could affect our estimates. However, based on a previous study by the NCHS (Arias et al., 2016), we estimate that deaths to the foreign-born population are underestimated by only 0.5 %, so we are confident none of our main conclusions would change. A third limitation is the use of single race data rather than ‘bridged’ race data due the unavailability of ‘bridged’ race population estimates in 2021 and 2022. We tested the sensitivity of our results in 2017–2019 and 2020 to the use of “bridged” rather than single race data and found no substantive changes in our main findings. Obtaining consistent population estimates for 2017–2022 also proved challenging because of changes in the race coding in Census-based data products in 2020 which produced a large increase in the multi-race population, and a discontinuity between postcensal population estimates in 2019 and the 2020 Census population estimates (Arias et al., 2025). We addressed these issues by combining ACS and Census data as outlined in the Supplemental Material. While we cannot rule out the possibility of remaining inaccuracies, our estimates of race and Hispanic-origin US life tables closely match the official ones (NVSS, 2025).

### 6.2. Conclusions

In 2020 the COVID-19 pandemic caused a large decline in US life expectancy among all foreign-born and US-born populations. Except for US-born Asian men and women, no group had recovered their pre-pandemic life-expectancy in 2022. All foreign-born subgroups experienced larger increases in mortality than their US-born counterparts in 2020. Even though this pattern reversed in 2021, only foreign-born Black and White men had recovered their advantage relative to their US-born counterparts in 2022. Despite the incomplete recovery of the foreign-born mortality advantage, the foreign-born populations continued to make important contributions to US life expectancy throughout the pandemic. These results demonstrate that US life expectancy would have been even lower and have declined further in the absence of the foreign-born mortality advantage. At the same time these contributions varied by race and Hispanic origin. As the US foreign-born population continues to grow increasingly diverse (National Academies of Sciences, Engineering, and Medicine, 2016), it is important to disaggregate mortality estimates by nativity, race, and Hispanic origin. In this paper we present the first estimates of life expectancies by nativity, race, and Hispanic-origin and their contributions to US life expectancy for the pandemic period, highlighting the diverging trends in US mortality across these key dimensions.

## Supplementary Material

Appendix

Appendix A. Supplementary data

Supplementary data to this article can be found online at https://doi.org/10.1016/j.socscimed.2025.118191.

## Figures and Tables

**Table 1:** Population by nativity, race, Hispanic origin, and sex (2017–2022).

	Males					Females				
	Foreign-Born			US-Born		Foreign-Born			US-Born	
	Population	%FB	% of Total	Population	% of Total	Population	%FB	% of Total	Population	% of Total
Asian	5,939,489	64.53	25.25	3,264,296	2.34	6,905,070	69.02	28.00	3,098,787	2.18
Black	2,131,411	10.75	9.06	17,699,920	12.71	2,290,472	10.64	9.29	19,232,440	13.56
Hispanic	10,471,598	33.85	44.51	20,465,858	14.70	10,155,957	33.65	41.18	20,028,793	14.12
White	4,515,755	4.63	19.20	93,100,498	66.86	4,813,332	4.84	19.51	94,716,390	66.77
Other or Multiple Race	465,546	8.98	1.98	4,721,300	3.39	500,348	9.47	2.03	4,784,783	3.37
Total	23,523,800	14.45	100.00	139,251,873	100.00	24,665,179	14.81	100.00	141,861,191	100.00

Notes: The numbers in the “% FB” column refer to the percentage of the population in the respective race/ethnicity and sex groups that are foreign born. The numbers in the “% of Total FB” columns refer to the proportion of the total foreign-born population represented by the race and Hispanic-origin groups. The numbers in the % Total USB column refer to the proportion of the total US-born population represented by the race and Hispanic-origin groups.

Source: American Community Survey 1-Year Files 2017–2022 and Census Bureau postcensal population estimates.

**Table 2:** Life expectancy by nativity, race and Hispanic-origin, 2017–2022.

	Females				Males			
	2017–2019	2020	2021	2022	2017–2019	2020	2021	2022
Panel A – Life expectancy by Nativity								
Foreign-born	84.8	82.3	82.7	83.6	80.7	76.5	77.2	78.7
US-born	79.9	78.4	77.9	78.7	74.9	73.0	72.1	73.3
Total	80.6	79.2	78.7	79.5	75.8	73.6	72.9	74.2
Foreign-born – US-Born	4.9	3.9	4.8	4.9	5.8	3.5	5.1	5.4
Panel B – Life expectancy by Nativity, Race and Hispanic-Origin								
Asian								
Foreign-born	86.8	85.0	85.5	86.0	83.2	80.4	80.9	81.8
US-born	86.7	87.0	86.1	87.1	82.2	81.6	80.6	82.2
Foreign-born – US-born	0.1	−2.0	−0.6	−1.1	1.0	−1.1	0.3	−0.4
Black								
Foreign-born	84.2	79.8	81.5	82.5	80.5	74.7	76.5	78.4
US-born	77.0	74.3	73.8	75.4	70.2	66.7	66.1	67.6
Foreign-born – US-born	7.2	5.5	7.8	7.1	10.3	8.0	10.3	10.8
Hispanic								
Foreign-born	84.8	81.4	81.7	83.3	80.5	74.4	75.0	77.5
US-born	82.2	80.0	79.5	81.1	76.8	73.9	73.1	75.3
Foreign-born – US-born	2.6	1.4	2.2	2.3	3.7	0.5	1.9	2.2
White								
Foreign-born	83.5	82.1	82.3	82.5	79.4	77.1	77.6	78.2
US-born	80.1	79.0	78.4	79.1	75.4	73.9	73.0	74.1
Foreign-born – US-born	3.4	3.1	3.9	3.4	4.1	3.1	4.6	4.1

Source: Authors calculations. Confidence intervals are shown in the Supplementary Material (Table S1).

**Table 3:** Contributions of foreign-born residents to US life expectancy by age group and cause of death, 2017–2022.

	Females				Males			
	2017–2019	2020	2021	2022	2017–2019	2020	2021	2022
Panel A – Foreign-born contribution to US life expectancy by age group								
Ages 1–24	0.01	<0.01	0.01	0.01	0.02	0.01	0.02	0.01
	(1 %)	(2 %)	(2 %)	(1 %)	(2 %)	(2 %)	(2 %)	(1 %)
Ages 25–64	0.43	0.41	0.52	0.5	0.58	0.44	0.59	0.6
	(57 %)	(66 %)	(66 %)	(63 %)	(66 %)	(77 %)	(73 %)	(70 %)
Ages 65 and above	0.31	0.21	0.26	0.29	0.28	0.12	0.2	0.25
	(41 %)	(33 %)	(33 %)	(36 %)	(32 %)	(20 %)	(25 %)	(29 %)
All ages	0.76	0.63	0.79	0.80	0.87	0.57	0.81	0.86
Panel B – Foreign-born contribution to US life expectancy by cause of death								
Natural (exc. COVID-19)	0.60	0.51	0.58	0.58	0.58	0.48	0.53	0.54
	(79 %)	(81 %)	(73 %)	(73 %)	(66 %)	(84 %)	(66 %)	(63 %)
External	0.16	0.17	0.18	0.18	0.29	0.21	0.32	0.30
	(21 %)	(27 %)	(23 %)	(23 %)	(24 %)	(37 %)	(40 %)	(35 %)
COVID-19	0.00	−0.05	0.04	0.03	0.00	−0.12	−0.05	0.02
	(0 %)	(−8 %)	(5 %)	(4 %)	(0 %)	(−20 %)	(−6 %)	(2 %)
All causes	0.76	0.63	0.79	0.80	0.87	0.57	0.81	0.86

Notes: The numbers outside of parentheses in Panel A and Panel B refer to contributions to the US life expectancy in years. The percentages in parentheses refer to the proportional contribution of age groups (Panel A) and causes of death (Panel B).

Source: Authors calculations.

## Data Availability

Replication materials for the article, including R code needed to replicate the analyses in the paper and instructions on how to obtain the data we cannot share directly, are available in an online repository at https://osf.io/fnws9/
