# Supplementary material for: US-born and foreign-born life expectancy by race and Hispanic origin before and during the COVID-19 pandemic in the United States
Source: Soc Sci Med. Author manuscript; Available in PMC 2025 Sep 1. (PMC12230979; doi:10.1016/j.socscimed.2025.118191)
Supplement: Appendix [file NIHMS2092959-supplement-Appendix.docx]

Supplementary Material for: US-Born and Foreign-born Life Expectancy by Race and Hispanic Origin before and during the COVID-19 Pandemic in the United States

Table S1: Life Expectancies at Age 1 by Nativity, Race, Hispanic-Origin, Sex, and Year

|  | Females | | | | Males | | | |
| --- | --- | --- | --- | --- | --- | --- | --- | --- |
| Year | Foreign-Born | US-Born | Total | Foreign-Born  Advantage | Foreign-Born | US-Born | Total | Foreign-Born  Advantage |
| Asian | | | | | | | | |
| 2017-2019 | 86.8 (86.4, 87.3) | 86.7 (84.5, 88.3) | 86.8 | 0.2 (-1.9, 2.8) | 83.2 (82.7, 83.6) | 82.2 (80.1, 83.9) | 82.9 | 1.0 (-1.2, 3.5) |
| 2020 | 85.0 (84.8, 85.1) | 87.0 (86.3, 87.5) | 85.2 | -2.0 (-2.7, -1.2) | 80.4 (80.3, 80.6) | 81.6 (80.7, 82.3) | 80.5 | -1.1 (-2.1, -0.2) |
| 2021 | 85.5 (85.3, 85.7) | 86.1 (84.8, 87.2) | 85.5 | -0.6 (-1.9, 1.0) | 80.9 (80.6, 81.2) | 80.6 (79.1, 82.0) | 80.8 | 0.3 (-1.3, 2.1) |
| 2022 | 86.0 (85.8, 86.2) | 87.1 (86.0, 88.0) | 86.1 | -1.1 (-2.3, 0.2) | 81.8 (81.6, 82.1) | 82.2 (80.9, 83.4) | 81.8 | -0.4 (-1.8, 1.2) |
| Black | | | | | | | | |
| 2017-2019 | 84.2 (82.5, 85.7) | 77.0 (76.8, 77.2) | 77.8 | 7.2 (5.3, 8.9) | 80.5 (78.5, 82.5) | 70.2 (70.0, 70.4) | 71.3 | 10.3 (8.1, 12.5) |
| 2020 | 79.8 (78.4, 80.9) | 74.3 (74.1, 74.5) | 74.9 | 5.5 (3.9, 6.8) | 74.7 (73.2, 75.8) | 66.7 (66.4, 66.9) | 67.6 | 8.0 (6.3, 9.4) |
| 2021 | 81.5 (79.6, 83.2) | 73.8 (73.6, 74.0) | 74.6 | 7.8 (5.6, 9.7) | 76.5 (74.5, 78.4) | 66.1 (65.8, 66.4) | 67.3 | 10.3 (8.0, 12.5) |
| 2022 | 82.5 (80.2, 84.4) | 75.4 (75.1, 75.7) | 76.2 | 7.1 (4.5, 9.3) | 78.4 (76.5, 80.3) | 67.6 (67.3, 67.9) | 68.8 | 10.8 (8.6, 12.9) |
| Hispanic | | | | | | | | |
| 2017-2019 | 84.8 (83.9, 85.6) | 82.2 (81.1, 83.2) | 83.6 | 2.6 (0.7, 4.5) | 80.5 (79.6, 81.2) | 76.8 (75.8, 77.7) | 78.8 | 3.7 (2.0, 5.4) |
| 2020 | 81.4 (80.9, 81.8) | 80.0 (79.5, 80.5) | 80.7 | 1.4 (0.5, 2.3) | 74.4 (73.9, 74.8) | 73.9 (73.5, 74.4) | 74.2 | 0.5 (-0.5, 1.4) |
| 2021 | 81.7 (81.2, 82.1) | 79.5 (78.9, 80.0) | 80.6 | 2.2 (1.2, 3.1) | 75.0 (74.5, 75.5) | 73.1 (72.6, 73.6) | 74.2 | 1.9 (0.8, 3.0) |
| 2022 | 83.3 (82.9, 83.8) | 81.1 (80.5, 81.6) | 82.3 | 2.3 (1.3, 3.3) | 77.5 (76.9, 78.0) | 75.3 (74.7, 75.7) | 76.5 | 2.2 (1.1, 3.3) |
| White | | | | | | | | |
| 2017-2019 | 83.5 (82.9, 84.1) | 80.1 (80.1, 80.1) | 80.3 | 3.4 (2.8, 4.1) | 79.4 (78.7, 80.1) | 75.4 (75.3, 75.4) | 75.6 | 4.1 (3.3, 4.8) |
| 2020 | 82.1 (81.7, 82.4) | 79.0 (79.0, 79.0) | 79.1 | 3.1 (2.8, 3.4) | 77.1 (76.6, 77.5) | 73.9 (73.9, 74.0) | 74.1 | 3.1 (2.7, 3.6) |
| 2021 | 82.3 (81.8, 82.9) | 78.4 (78.4, 78.5) | 78.7 | 3.9 (3.3, 4.5) | 77.6 (76.9, 78.3) | 73.0 (73.0, 73.1) | 73.3 | 4.6 (3.8, 5.3) |
| 2022 | 82.5 (81.9, 83.0) | 79.1 (79.1, 79.1) | 79.3 | 3.4 (2.8, 3.9) | 78.2 (77.5, 78.8) | 74.1 (74.1, 74.1) | 74.3 | 4.1 (3.4, 4.7) |
| Total | | | | | | | | |
| 2017-2019 | 84.8 (84.0, 85.4) | 79.9 (79.7, 80.0) | 80.6 | 4.9 (4.1, 5.7) | 80.7 (80.0, 81.4) | 74.9 (74.8, 75.0) | 75.8 | 5.8 (4.9, 6.7) |
| 2020 | 82.3 (81.9, 82.7) | 78.4 (78.4, 78.5) | 79.1 | 3.9 (3.4, 4.3) | 76.5 (76.0, 76.9) | 73.0 (72.9, 73.1) | 73.6 | 3.5 (2.9, 4.0) |
| 2021 | 82.7 (82.2, 83.2) | 77.9 (77.8, 78.0) | 78.7 | 4.8 (4.2, 5.4) | 77.2 (76.5, 77.8) | 72.1 (72.0, 72.2) | 72.9 | 5.1 (4.3, 5.8) |
| 2022 | 83.6 (83.1, 84.1) | 78.7 (78.6, 78.9) | 79.5 | 4.9 (4.2, 5.5) | 78.7 (78.1, 79.3) | 73.3 (73.2, 73.5) | 74.2 | 5.4 (4.6, 6.1) |

*Source: Authors’ calculations based on data from the National Vital Statistics Systems and the American Community Survey.*

*Notes: Central estimates are presented outside parentheses with upper and lower bounds reflecting uncertainty in the proportion of the population that is foreign-born reported inside the parentheses. Life expectancy estimates for the total population include multi-race individuals.*

Table S2: Contributions of Foreign-Born Residents to US Life expectancy by Race and Hispanic Origin, 2017-2022.

|  | Females | | Males | |
| --- | --- | --- | --- | --- |
| Year | Contribution | % of Total | Contribution | % of Total |
| Asian | | | | |
| 2017-2019 | 0.268 | 35.35 | 0.290 | 33.34 |
| 2020 | 0.276 | 44.06 | 0.288 | 50.38 |
| 2021 | 0.325 | 40.94 | 0.337 | 41.60 |
| 2022 | 0.311 | 38.98 | 0.330 | 38.38 |
| Black | | | | |
| 2017-2019 | 0.049 | 6.46 | 0.063 | 7.29 |
| 2020 | 0.022 | 3.51 | 0.032 | 5.66 |
| 2021 | 0.049 | 6.21 | 0.067 | 8.25 |
| 2022 | 0.051 | 6.42 | 0.073 | 8.53 |
| Hispanic | | | | |
| 2017-2019 | 0.288 | 38.09 | 0.338 | 38.88 |
| 2020 | 0.198 | 31.57 | 0.112 | 19.52 |
| 2021 | 0.254 | 32.01 | 0.208 | 25.63 |
| 2022 | 0.297 | 37.23 | 0.281 | 32.65 |
| White | | | | |
| 2017-2019 | 0.136 | 17.92 | 0.157 | 18.08 |
| 2020 | 0.125 | 20.03 | 0.134 | 23.37 |
| 2021 | 0.155 | 19.47 | 0.187 | 23.10 |
| 2022 | 0.125 | 15.70 | 0.158 | 18.35 |
| Other or Multiple Race | | | | |
| 2017-2019 | 0.016 | 2.18 | 0.021 | 2.40 |
| 2020 | 0.005 | 0.84 | 0.006 | 1.08 |
| 2021 | 0.011 | 1.37 | 0.012 | 1.42 |
| 2022 | 0.013 | 1.68 | 0.018 | 2.10 |
| Total | | | | |
| 2017-2019 | 0.757 | 100.00 | 0.869 | 100.00 |
| 2020 | 0.626 | 100.00 | 0.572 | 100.00 |
| 2021 | 0.794 | 100.00 | 0.811 | 100.00 |
| 2022 | 0.798 | 100.00 | 0.859 | 100.00 |

*Source: Authors’ calculations based on data from the National Vital Statistics Systems and the American Community Survey.*

Table S3: Contributions of Foreign-Born Residents to US Life expectancy by Race, Hispanic-Origin, Sex, Broad Age-Groups and Year

|  | Females | | | | Males | | | |
| --- | --- | --- | --- | --- | --- | --- | --- | --- |
| Year | 1-24 | 25-64 | 65+ | All Ages | 1-24 | 25-64 | 65+ | All Ages |
| Asian | | | | | | | | |
| 2017-2019 | <0.01 (2%) | 0.14 (54%) | 0.12 (44%) | 0.268 | <0.01 (3%) | 0.17 (60%) | 0.11 (37%) | 0.290 |
| 2020 | <0.01 (2%) | 0.16 (59%) | 0.11 (39%) | 0.276 | <0.01 (3%) | 0.19 (66%) | 0.09 (31%) | 0.288 |
| 2021 | <0.01 (2%) | 0.19 (60%) | 0.13 (39%) | 0.325 | <0.01 (3%) | 0.23 (67%) | 0.1 (30%) | 0.337 |
| 2022 | <0.01 (2%) | 0.18 (57%) | 0.13 (42%) | 0.311 | <0.01 (3%) | 0.21 (64%) | 0.11 (33%) | 0.330 |
| Black | | | | | | | | |
| 2017-2019 | <0.01 (2%) | 0.03 (57%) | 0.02 (41%) | 0.049 | <0.01 (2%) | 0.05 (74%) | 0.02 (24%) | 0.063 |
| 2020 | <0.01 (4%) | 0.02 (97%) | <0.01 (0%) | 0.022 | <0.01 (4%) | 0.04 (117%) | <0.01 (-21%) | 0.032 |
| 2021 | <0.01 (3%) | 0.04 (74%) | 0.01 (24%) | 0.049 | <0.01 (2%) | 0.06 (87%) | <0.01 (10%) | 0.067 |
| 2022 | <0.01 (2%) | 0.04 (69%) | 0.02 (30%) | 0.051 | <0.01 (1%) | 0.06 (81%) | 0.01 (18%) | 0.073 |
| Hispanic | | | | | | | | |
| 2017-2019 | <0.01 (1%) | 0.18 (64%) | 0.1 (35%) | 0.288 | <0.01 (1%) | 0.25 (73%) | 0.09 (26%) | 0.338 |
| 2020 | <0.01 (1%) | 0.15 (77%) | 0.04 (22%) | 0.198 | <0.01 (0%) | 0.11 (101%) | <0.01 (0%) | 0.112 |
| 2021 | <0.01 (1%) | 0.19 (75%) | 0.06 (23%) | 0.254 | <0.01 (1%) | 0.17 (84%) | 0.03 (15%) | 0.208 |
| 2022 | <0.01 (1%) | 0.2 (69%) | 0.09 (30%) | 0.297 | <0.01 (-1%) | 0.21 (76%) | 0.07 (25%) | 0.281 |
| White | | | | | | | | |
| 2017-2019 | <0.01 (1%) | 0.07 (50%) | 0.07 (49%) | 0.136 | <0.01 (1%) | 0.1 (62%) | 0.06 (37%) | 0.157 |
| 2020 | <0.01 (1%) | 0.07 (58%) | 0.05 (41%) | 0.125 | <0.01 (2%) | 0.1 (74%) | 0.03 (25%) | 0.134 |
| 2021 | <0.01 (1%) | 0.09 (61%) | 0.06 (37%) | 0.155 | <0.01 (2%) | 0.13 (67%) | 0.06 (31%) | 0.187 |
| 2022 | <0.01 (1%) | 0.08 (62%) | 0.05 (37%) | 0.125 | <0.01 (2%) | 0.11 (69%) | 0.05 (29%) | 0.158 |
| Other or Multiple Race | | | | | | | | |
| 2017-2019 | <0.01 (2%) | <0.01 (48%) | <0.01 (50%) | 0.016 | <0.01 (5%) | 0.01 (55%) | <0.01 (39%) | 0.021 |
| 2020 | <0.01 (6%) | <0.01 (40%) | <0.01 (54%) | 0.005 | <0.01 (12%) | <0.01 (60%) | <0.01 (28%) | 0.006 |
| 2021 | <0.01 (6%) | <0.01 (55%) | <0.01 (40%) | 0.011 | <0.01 (9%) | <0.01 (49%) | <0.01 (42%) | 0.012 |
| 2022 | <0.01 (5%) | <0.01 (45%) | <0.01 (50%) | 0.013 | <0.01 (7%) | <0.01 (53%) | <0.01 (40%) | 0.018 |
| Total | | | | | | | | |
| 2017-2019 | 0.01 (1%) | 0.43 (57%) | 0.31 (41%) | 0.757 | 0.02 (2%) | 0.58 (66%) | 0.28 (32%) | 0.869 |
| 2020 | <0.01 (2%) | 0.41 (66%) | 0.21 (33%) | 0.626 | 0.01 (2%) | 0.44 (77%) | 0.12 (20%) | 0.572 |
| 2021 | 0.01 (2%) | 0.52 (66%) | 0.26 (33%) | 0.794 | 0.02 (2%) | 0.59 (73%) | 0.2 (25%) | 0.811 |
| 2022 | 0.01 (1%) | 0.5 (63%) | 0.29 (36%) | 0.798 | 0.01 (1%) | 0.6 (70%) | 0.25 (29%) | 0.859 |

*Source: Authors’ calculations based on data from the National Vital Statistics Systems and the American Community Survey.*

*Notes: The numbers outside of parentheses are contributions to the US life expectancy in years. The percentages in parentheses refer to the proportional contribution of the different age groups to the contribution of each race and Hispanic-origin group by sex (i.e. rows under the Females and Males heading each sum to 100%).*

Table S4: Contributions of Foreign-born Residents to US Life Expectancy by Race, Hispanic Origin and Cause of Death

|  | Females | | | | Males | | | |
| --- | --- | --- | --- | --- | --- | --- | --- | --- |
| Year | Natural  (exc. COVID-19) | External | COVID-19 | All Causes | Natural  (exc. COVID-19) | External | COVID-19 | All Causes |
| Asian | | | | | | | | |
| 2017-2019 | 0.219 | 0.048 | 0.000 | 0.268 | 0.194 | 0.096 | 0.000 | 0.290 |
| 2020 | 0.208 | 0.055 | 0.012 | 0.276 | 0.178 | 0.109 | 0.001 | 0.288 |
| 2021 | 0.226 | 0.064 | 0.036 | 0.325 | 0.191 | 0.120 | 0.027 | 0.337 |
| 2022 | 0.233 | 0.062 | 0.016 | 0.311 | 0.199 | 0.117 | 0.013 | 0.330 |
| Black | | | | | | | | |
| 2017-2019 | 0.034 | 0.015 | 0.000 | 0.049 | 0.036 | 0.028 | 0.000 | 0.063 |
| 2020 | 0.011 | 0.015 | -0.004 | 0.022 | 0.020 | 0.025 | -0.013 | 0.032 |
| 2021 | 0.032 | 0.016 | 0.001 | 0.049 | 0.033 | 0.031 | 0.003 | 0.067 |
| 2022 | 0.032 | 0.017 | 0.002 | 0.051 | 0.038 | 0.032 | 0.003 | 0.073 |
| Hispanic | | | | | | | | |
| 2017-2019 | 0.216 | 0.072 | 0.000 | 0.288 | 0.216 | 0.122 | 0.000 | 0.338 |
| 2020 | 0.191 | 0.072 | -0.066 | 0.198 | 0.189 | 0.028 | -0.106 | 0.112 |
| 2021 | 0.207 | 0.072 | -0.025 | 0.254 | 0.180 | 0.119 | -0.092 | 0.208 |
| 2022 | 0.212 | 0.078 | 0.006 | 0.297 | 0.189 | 0.093 | -0.002 | 0.281 |
| White | | | | | | | | |
| 2017-2019 | 0.116 | 0.020 | 0.000 | 0.136 | 0.116 | 0.041 | 0.000 | 0.157 |
| 2020 | 0.095 | 0.020 | 0.010 | 0.125 | 0.087 | 0.044 | 0.003 | 0.134 |
| 2021 | 0.106 | 0.025 | 0.024 | 0.155 | 0.123 | 0.048 | 0.016 | 0.187 |
| 2022 | 0.094 | 0.024 | 0.007 | 0.125 | 0.105 | 0.048 | 0.005 | 0.158 |
| Other or Multiple Race | | | | | | | | |
| 2017-2019 | 0.013 | 0.003 | 0.000 | 0.016 | 0.014 | 0.007 | 0.000 | 0.021 |
| 2020 | 0.004 | 0.002 | -0.001 | 0.005 | 0.004 | 0.003 | -0.001 | 0.006 |
| 2021 | 0.007 | 0.003 | 0.000 | 0.011 | 0.006 | 0.005 | 0.000 | 0.012 |
| 2022 | 0.010 | 0.003 | 0.000 | 0.013 | 0.011 | 0.007 | 0.001 | 0.018 |
| Total | | | | | | | | |
| 2017-2019 | 0.599 | 0.158 | 0.000 | 0.757 | 0.576 | 0.293 | 0.000 | 0.869 |
| 2020 | 0.509 | 0.165 | -0.048 | 0.626 | 0.478 | 0.210 | -0.115 | 0.572 |
| 2021 | 0.578 | 0.181 | 0.036 | 0.794 | 0.533 | 0.323 | -0.045 | 0.811 |
| 2022 | 0.582 | 0.184 | 0.032 | 0.798 | 0.542 | 0.297 | 0.021 | 0.859 |

*Source: Authors’ calculations based on data from the National Vital Statistics Systems and the American Community Survey.*

Table S5: Population Distribution of Foreign-Born and US-Born Residents by Metro Status and Sex. US residents aged 18 and above (2017-2021).

|  | US-Born | | Foreign-Born | |
| --- | --- | --- | --- | --- |
| Metro Category | Population | % of Total | Population | % of Total |
| Female | | | | |
| Large Central Metro | 29,300,297 | 26.98 | 11,101,928 | 51.18 |
| Large Fringe Metro | 27,206,258 | 25.06 | 5,641,050 | 26.01 |
| Medium or Small Metro | 34,965,199 | 32.20 | 4,137,229 | 19.07 |
| Nonmetro | 17,113,405 | 15.76 | 810,869 | 3.74 |
| Male | | | | |
| Large Central Metro | 27,822,763 | 26.60 | 10,485,830 | 50.90 |
| Large Fringe Metro | 26,016,415 | 24.87 | 5,309,953 | 25.78 |
| Medium or Small Metro | 33,677,365 | 32.19 | 3,945,088 | 19.15 |
| Nonmetro | 17,098,482 | 16.34 | 859,275 | 4.17 |

*Source: 2017-2021 ACS five-year files accessed through IPUMS National Historical Geographic Information System* (Manson et al. 2023)*.*

Table S6: Population Distribution of Foreign-Born and US-Born Residents by Census Division and Sex. US residents aged 18 and above (2017-2021).

|  | US-Born | | Foreign-Born | |
| --- | --- | --- | --- | --- |
| Census Division | Population | % of Total | Population | % of Total |
| Female | | | | |
| East North Central | 16,968,405 | 15.63 | 1,741,952 | 8.03 |
| East South Central | 7,369,063 | 6.79 | 349,057 | 1.61 |
| Middle Atlantic | 13,460,283 | 12.40 | 3,730,394 | 17.20 |
| Mountain | 8,112,908 | 7.47 | 1,304,319 | 6.01 |
| New England | 5,218,582 | 4.81 | 1,001,120 | 4.62 |
| Pacific | 14,741,509 | 13.58 | 6,096,380 | 28.11 |
| South Atlantic | 22,195,904 | 20.44 | 4,323,261 | 19.93 |
| West North Central | 7,714,710 | 7.10 | 599,049 | 2.76 |
| West South Central | 12,803,795 | 11.79 | 2,545,544 | 11.74 |
| Male | | | | |
| East North Central | 16,222,813 | 15.51 | 1,752,852 | 8.51 |
| East South Central | 6,851,980 | 6.55 | 363,649 | 1.77 |
| Middle Atlantic | 12,742,403 | 12.18 | 3,424,019 | 16.62 |
| Mountain | 8,195,451 | 7.83 | 1,227,210 | 5.96 |
| New England | 4,931,360 | 4.71 | 927,960 | 4.50 |
| Pacific | 14,906,386 | 14.25 | 5,684,936 | 27.60 |
| South Atlantic | 20,885,241 | 19.96 | 4,033,324 | 19.58 |
| West North Central | 7,545,371 | 7.21 | 609,338 | 2.96 |
| West South Central | 12,334,020 | 11.79 | 2,576,858 | 12.51 |

*Source: 2017-2021 ACS five-year files accessed through IPUMS National Historical Geographic Information System* (Manson et al. 2023)*.*

Figure S1: Ratios of Age-Specific Mortality-Rates between US-Born Residents by group and Foreign-Born Residents Belonging to the Same group (Panel A) and Life Expectancies By Nativity, Sex, and Race Ethnicity (Panel B).


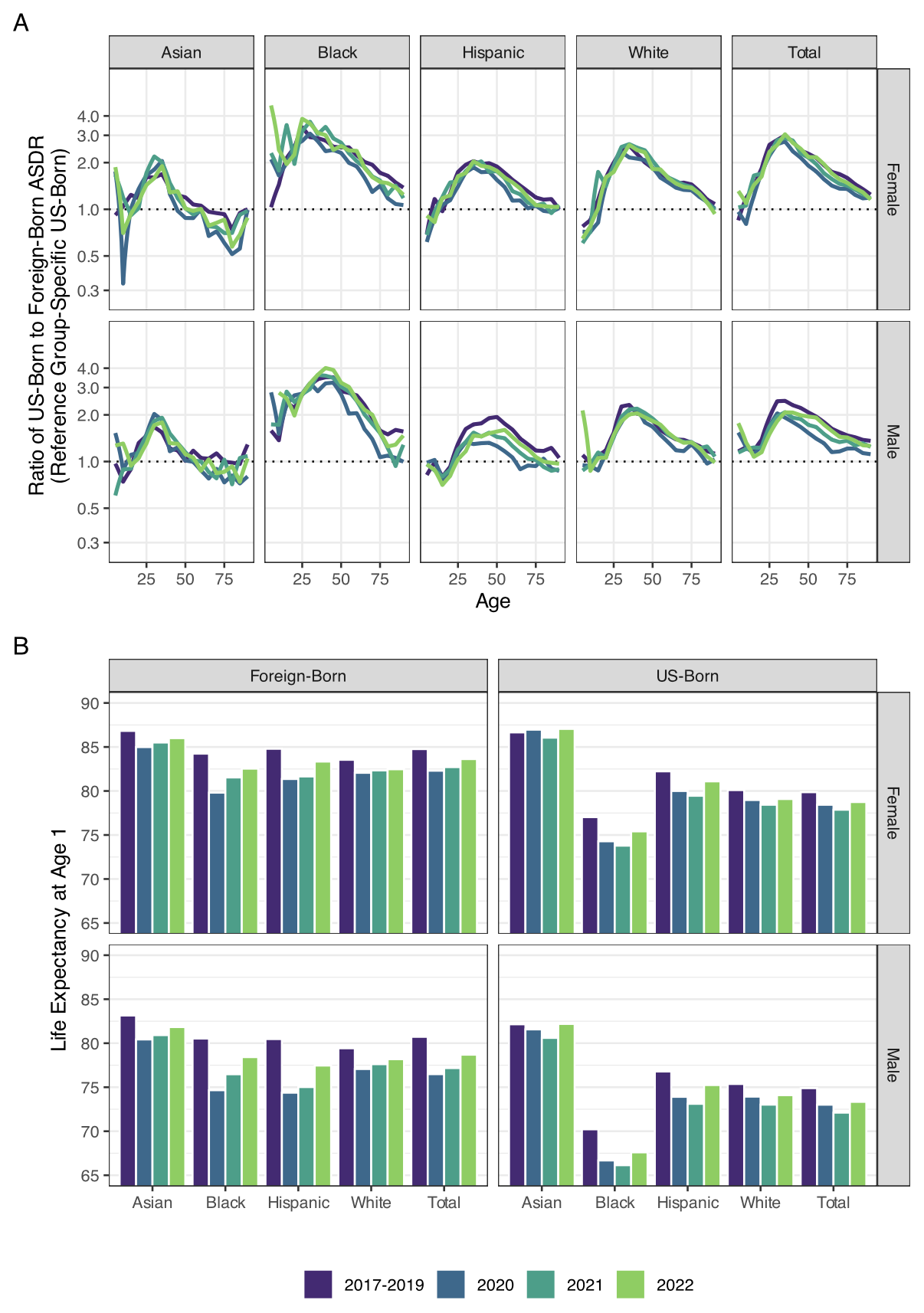


Figure S2: Contribution of Foreign-Born Residents to US Life Expectancy by Race, Hispanic Origin, Sex, Year, and by Cause-of-Death (detailed).


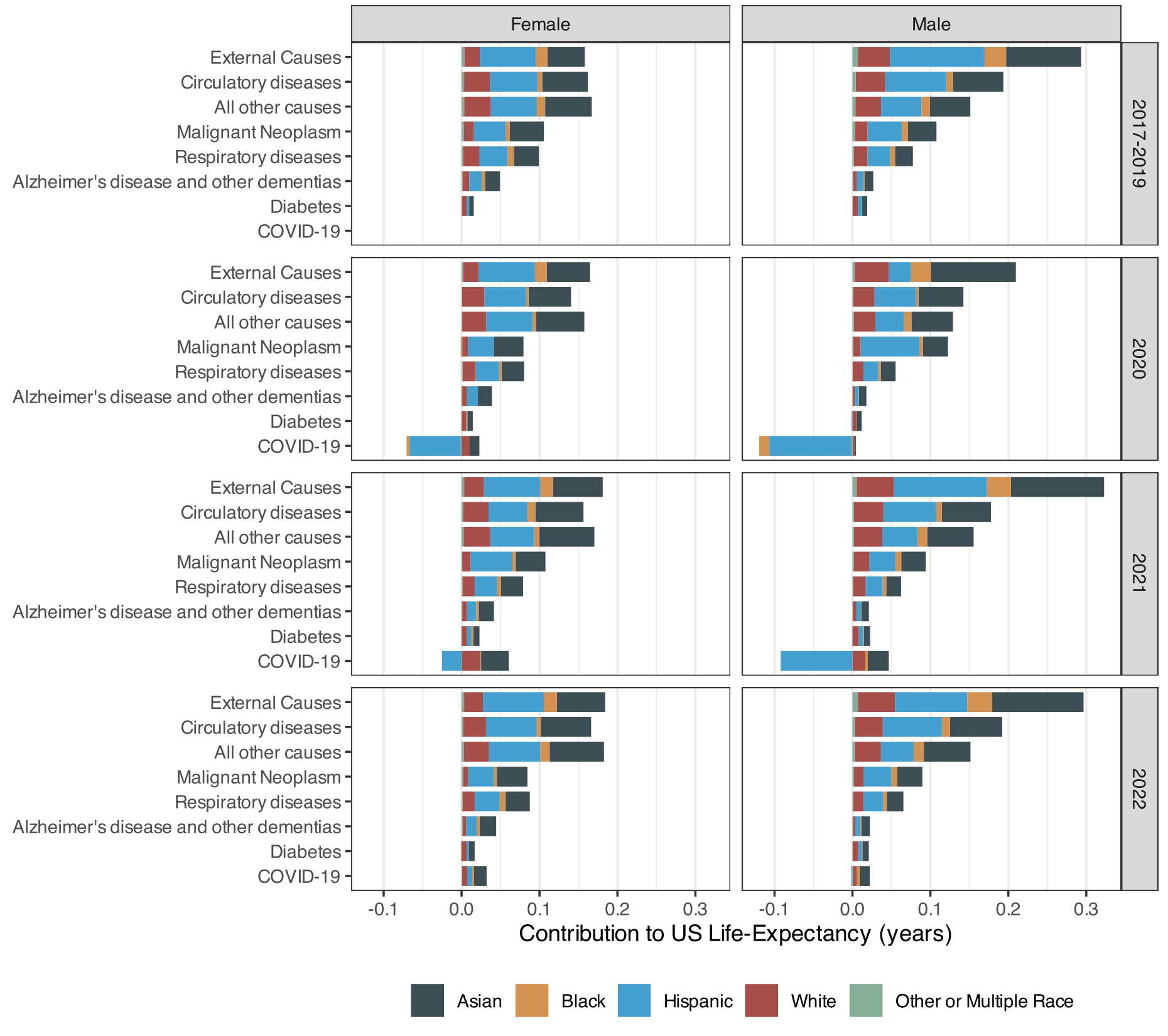


*Notes: Contributions are presented as stacked bars so that the area in each graph equals to the contributions of foreign-born residents by race and Hispanic-origin and each bar equals the total cause-specific contribution of all foreign-born residents.*

## Details on Population Estimates and Life Table Construction

We began by using US resident population counts by sex, age, race, Hispanic origin, and place of birth for 2017-2022 from the 1-year American Community Survey (ACS) files (Ruggles et al. 2022). However, changes in the ACS questionnaire and in the coding of write-in responses in the race questions beginning in 2020, substantially altered the proportion of respondents classified as more than one race (Arias et al. 2025; Jones et al. 2021). The population count for the “Two or More Races” group climbed from 9.3 million in 2019 to 15.1 million in 2020, a 62.9% increase. This increase should be compared with a less than 1% increase from 2018 to 2019. The Census Bureau’s postcensal population estimates in contrast show a 2.4% increase from 2018 to 2019, and a 4.9% increase from 2019 to 2020 (US Census Bureau 2020, 2023). All racial and Hispanic-origin groups appear to have contributed to the increase in the “Two or More Races” group in the ACS. At the same time, overall population trends in the Census estimates show a large increase in population between 2019 and 2020 (US Census Bureau 2020, 2023). In the absence of revised postcensal population counts between 2010 and 2020, the ACS provides a more consistent series of yearly population totals, whereas the Census postcensal estimates provide a more consistent estimates by race/ethnicity, which do not appear to be affected by the change in the race question in the 2020 Census and the ACS.

### Adjustment Procedure

To eliminate the distortions in the proportion of the population by race/ethnicity introduced by the ACS question change, we developed the following procedure. We start with the Census Bureau’s postcensal population counts by year (2017-2022), five-year age groups, sex, race, and Hispanic origin. To our knowledge these counts are unaffected by the ACS question change. A first issue is that ages 0-4 are collapsed into a single age group and the open-ended age interval begins at age 85. Because we want our age groups to be <1, 1-4, 5-9, 10-14, …, 90+, we first compute the proportion of the population in the age group 0-4 who are less than age 1, and the proportion in the age group 85+ who are aged 85-89 by year, sex, and race/ethnicity from the ACS data (1-year files 2017-2022). We use these proportions to distribute the population of age-group 0-4 into age groups <1 and 1-4 and to distribute the population of the age group 85+ into age groups 85-89 and 90+. For the next step, we compute the proportion of foreign-born residents by year, sex, age, race, and Hispanic origin in the ACS and apply these proportions to the age-specific postcensal population estimates to allocate the population in each cell into foreign-born and US-born population. Finally, we multiply population estimates in all cells with year-specific adjustment ratios that rescale total population to the ACS rather than the postcensal yearly totals. This procedure ensures that the distribution of the population by race and Hispanic origin is consistent with the postcensal Census estimates. It eliminates the distortions in the ACS data caused by changes in the race question and coding of write-in responses. It also simultaneously rescales the population totals to the ACS estimates, eliminating the 2019-2020 discontinuity in the Census postcensal population estimates.

### Further Adjustments for Misclassification of Hispanic Origin and Race on Death Certificates

The official life tables by race and Hispanic origin for the United States adjust for Hispanic origin and race misclassification on death certificates using linked data from the National Longitudinal Mortality Study (NLSM), which links survey respondents in the Current Population Survey (CPS) to deaths in the National Death Index (NDI). The correction consists of multiplying death counts by age, sex, race, and Hispanic origin by a set of classification ratios which correct for under- or over-reporting of deaths produced by the misclassification of race or Hispanic origin on death certificates. These ratios for the 2019 life tables (Arias and Xu 2022a) are reported in Table A1 below. We were initially unsure about the use of these classification ratios because no distinction is made between US-born and foreign-born individuals, so that the same ratios must be applied to both groups. However, we found that applying the same ratios to both groups led to life tables closer to the official ones than those obtained ignoring misclassification. Finally, it is also important to note that while the main substantive conclusions do not change regardless of what population estimates we use, the precise numbers exhibit some variability. Furthermore, sensitivity analysis using bridged race data available for 2017-2020 confirm that the use of single race for Asian, Black, and White populations does not alter our substantive conclusions.

### Investigation of Misclassification of Place of Birth on Death Certificates

We are not aware of recent studies assessing directly the quality of place of birth reporting in deaths certificates. However, a recent NCHS study on the quality of race and Hispanic origin reporting (Arias et al. 2016) contains information on nativity as a binary indicator for deaths to foreign-born or US-born individuals. The study uses data from the National Longitudinal Mortality Study (NLMS) which links respondents of the Current Population Survey (CPS) to death records in the National Death Index (NDI) allowing the comparison on self-reported information in the CPS with that reported by funeral directors or medical examiners on death certificates. We use data from Supporting Table III, reporting death counts by race and Hispanic origin and by nativity using CPS and NDI information. As reported in Table A2, we find that without any adjustment for misclassification of race and Hispanic origin, the proportion of deaths to foreign-born individuals is overestimated by 2.3% for the Hispanic population, and underestimated by 1.6% for the White population, 2.4% for the Black population and 0.2% for the Asian and Pacific Islanders (API) population. For the total population, the proportion of deaths to foreign-born individuals is underestimated by 2.5%. We also proceeded to adjust the death counts for race and Hispanic origin misclassification. We did this by computing proportions of deaths by race and Hispanic origin according to CPS and NDI information and then computing ratios of these proportions (CPS/NDI) and multiplying deaths counts based on NDI information by these adjustment factors. This has the effect of giving deaths by race and Hispanic origin based on NDI information the same distribution by race and Hispanic origin as the counts based on CPS information. For example, the proportion of deaths to Hispanic individuals is 6.0% based on CPS information but 5.6% based on NDI information. To adjust for the underestimation of Hispanic deaths in the NDI data we multiply Hispanic death counts by 6.0/5.6=1.066. Having applied these adjustments, we find that the overall underestimation of the proportion of deaths in the foreign-born population based on NDI information is only 0.5%. The adjustment does not affect the nativity misclassification ratios by race and Hispanic origin because the same adjustment is applied to foreign-born and US-born deaths.

### Life Table Construction

To convert the observed mortality rates ${}_{n}M_{x}$ into probabilities of dying ${}_{n}q_{x}$, and to estimate abridged life tables, we follow a three-step procedure:

1. We graduate the observed ${}_{n}M_{x}$ rates for age groups 1-4, 5-9, …, 90+ into single-age rates using a one-dimensional smoother (Camarda 2012). This approach gives results similar to the Penalized Composite Link Model (Rizzi, Gampe, and Eilers 2015) but leads to life tables for the total population by sex closer to the official ones produced by the CDC (Arias and Xu 2022a, 2022b).
2. We extrapolate the single-age mortality rates to age 105 using a Gompertz model (Gompertz 1825; Riffe et al. 2019). For the extrapolation, we fit the Gompertz model on ages 85 to 90. While this is an unusual range, including all ages above 60, as in the default options of the DemoTools R package (Riffe et al. 2019), leads to life expectancies too high compared to the official life tables (Arias and Xu 2022b, 2022a). We then use standard life table methodology (Preston, Heuveline, and Guillot 2001) to construct a single-age or complete life table.
3. Finally, we extract the abridged life tables directly from the complete single-age life tables using the following identities:

$${}_{n}L_{x}=T_{x}-T_{x+n}$$

$${}_{n}d_{x}=l_{x}-l_{x+n}$$

$${}_{n}q_{x}=\frac{{}_{n}d_{x}}{l_{x}}$$

$${}_{n}a_{x}=\frac{{}_{n}L_{x}-n{\cdot l}_{x+n}}{{}_{n}d_{x}}$$

This three-step procedure can be considered an extension to other similar methods based on interpolating or graduating ${}_{n}M_{x}$ (Greville 1943; Keyfitz 1966; Keyfitz and Frauenthal 1975). We compared the results of our procedure with those produced using Greville’s (1943) formula and found that while the results were generally similar, ours were closer to the official life tables. See Section 3.2 of *Demography: Measuring and Modeling Population Processes* (Preston et al. 2001) or section 2.2 in *Applied Mathematical Demography* (Keyfitz and Caswell 2005) for a more in-depth discussion of different alternatives.

### Quantifying Uncertainty for Life Expectancies

In the construction of life tables we combine death record from the National Vital Statistics System that are considered complete, intercensal population estimates by year, sex, age, race, and Hispanic origin from the Census Bureau which are constructed based on birth and death records starting from estimates in a census year (2010 and 2020 for the period we examine), and survey-based estimates of the proportion of the population that is foreign-born by year, sex, age, race, and Hispanic origin, coming from the 1-year ACS files. While the first two sources can be considered to be without meaningful uncertainty given that they cover the entire US population, the proportions foreign-born which are based on the ACS survey sample carry uncertainty with them. We estimate the potential impact this uncertainty has on our estimated life expectancies with a four-step procedure:

1. We followed the instructions by IPUMS (IPUMS 2025)to create standard errors for each of the proportions US-born using the replicate weights provided by the ACS.
2. For each proportion $p$, we then use the calculated standard errors $p_{SE}$ to compute the 2.5^th^ ($p-1.96\cdot p_{SE}$) and 97.5^th^ ($p+1.96\cdot p_{SE}$) percentiles of its theoretical distribution under a Normal approximation.
3. We then use these two bounds to reassign the population by year, sex, age, race, and Hispanic origin into the US-born and foreign-born categories.
4. Finally, we re-estimate life tables for all groups first based on the original proportions, then based on the lower bound proportions and on the upper bound proportions.

The lower and upper bounds obtained through this procedure should not be strictly interpreted as 95% confidence intervals but rather as conservative estimates of the uncertainty surrounding each life expectancy. These bounds are derived under the assumption that the error in one proportion is independent of the errors in other proportions, which might not hold in practice. Ignoring the correlation between errors likely leads to intervals that are too wide, since we assume that all proportions are simultaneously wrong in the same direction, which is why we consider our bounds to be conservative.

Table A1: Classification Ratios from Table II in the Technical Appending to the National Vital Statistics System United States Life Tables for 2019

|  | Hispanic | | Non-Hispanic White | | Non-Hispanic Black | | Non-Hispanic Asian | |
| --- | --- | --- | --- | --- | --- | --- | --- | --- |
| Age | Male | Female | Male | Female | Male | Female | Male | Female |
| <1 | 1.0384 | 1.0263 | 0.9717 | 0.9926 | 1.0517 | 1.0408 | 0.7966 | 0.7552 |
| 1-4 | 0.9659 | 1.0299 | 1.0755 | 0.8770 | 0.9379 | 1.1751 | 0.8426 | 1.0000 |
| 5-9 | 0.9659 | 1.0299 | 1.0755 | 0.8770 | 0.9379 | 1.1751 | 0.8426 | 1.0000 |
| 10-14 | 0.9659 | 1.0299 | 1.0755 | 0.8770 | 0.9379 | 1.1751 | 0.8426 | 1.0000 |
| 15-19 | 0.9325 | 1.0604 | 1.0019 | 0.9869 | 1.0215 | 1.0343 | 1.4276 | 0.9721 |
| 20-24 | 0.9325 | 1.0604 | 1.0019 | 0.9869 | 1.0215 | 1.0343 | 1.4276 | 0.9721 |
| 25-29 | 1.0401 | 1.0232 | 1.0034 | 0.9994 | 0.9770 | 1.0008 | 1.0967 | 1.2648 |
| 30-34 | 1.0401 | 1.0232 | 1.0034 | 0.9994 | 0.9770 | 1.0008 | 1.0967 | 1.2648 |
| 35-39 | 1.0645 | 1.0066 | 0.9997 | 0.9951 | 1.0073 | 1.0048 | 1.0459 | 1.0125 |
| 40-44 | 1.0645 | 1.0066 | 0.9997 | 0.9951 | 1.0073 | 1.0048 | 1.0459 | 1.0125 |
| 45-49 | 1.0372 | 1.0953 | 0.9965 | 0.9976 | 1.0019 | 0.9982 | 1.1123 | 1.0113 |
| 50-54 | 1.0372 | 1.0953 | 0.9965 | 0.9976 | 1.0019 | 0.9982 | 1.1123 | 1.0113 |
| 55-59 | 1.0517 | 1.0659 | 0.9992 | 0.9997 | 0.9965 | 1.0046 | 1.0694 | 0.9784 |
| 60-64 | 1.0517 | 1.0659 | 0.9992 | 0.9997 | 0.9965 | 1.0046 | 1.0694 | 0.9784 |
| 65-69 | 1.0485 | 1.0072 | 0.9967 | 0.9966 | 1.0055 | 1.0070 | 1.0841 | 1.0850 |
| 70-74 | 1.0485 | 1.0072 | 0.9967 | 0.9966 | 1.0055 | 1.0070 | 1.0841 | 1.0850 |
| 75-79 | 1.0188 | 1.0196 | 1.0003 | 1.0004 | 1.0057 | 1.0058 | 1.0328 | 1.0281 |
| 80-84 | 1.0188 | 1.0196 | 1.0003 | 1.0004 | 1.0057 | 1.0058 | 1.0328 | 1.0281 |
| 85-89 | 1.0313 | 1.0137 | 1.0007 | 1.0009 | 1.0155 | 1.0086 | 0.9983 | 0.9944 |
| 90+ | 1.0509 | 1.0842 | 0.9995 | 1.0008 | 0.9872 | 0.9954 | 1.0238 | 0.9405 |

*Source: National Vital Statistics System United States Life Tables for 2019* (Arias and Xu 2022a)*.*

Table A2: Death Counts by Race and Hispanic origin and by Nativity and Proportion Foreign-Born Using Information from CPS and Death Certificates (NDI).

| Raw Data | | | | | | | |
| --- | --- | --- | --- | --- | --- | --- | --- |
|  | Deaths among US-Born | | Deaths among Foreign-Born | | Proportion Foreign-Born | | |
|  | CPS | Death Certificate | CPS | Death Certificate | CPS | Death Certificate | Ratio |
| Hispanic | 7,478 | 7,028 | 5,934 | 5,815 | 44.2% | 45.3% | 1.023 |
| White | 175,886 | 181,071 | 8,033 | 8,130 | 4.4% | 4.3% | 0.984 |
| Black | 21,263 | 21,269 | 528 | 515 | 2.4% | 2.4% | 0.976 |
| API | 1,824 | 1,787 | 1,922 | 1,874 | 51.3% | 51.2% | 0.998 |
| Total | 206,451 | 211,155 | 16,417 | 16,334 | 7.4% | 7.2% | 0.975 |
| Adjusted for Race and Hispanic Origin Misclassification | | | | | | | |
|  | Deaths among US-Born | | Deaths among Foreign-Born | | Proportion Foreign-Born | | |
|  | CPS | Death Certificate | CPS | Death Certificate | CPS | Death Certificate | Ratio |
| Hispanic | 7,478 | 7,492 | 5,934 | 6,199 | 44.2% | 45.3% | 1.023 |
| White | 175,886 | 180,377 | 8,033 | 8,099 | 4.4% | 4.3% | 0.984 |
| Black | 21,263 | 21,199 | 528 | 513 | 2.4% | 2.4% | 0.976 |
| API | 1,824 | 1,787 | 1,922 | 1,874 | 51.3% | 51.2% | 0.998 |
| Total | 206,451 | 210,855 | 16,417 | 16,685 | 7.4% | 7.3% | 0.995 |

*Source: Supporting Table III* (Arias et al. 2016)*.*

*Notes: The term Hispanic refers to individuals of Hispanic origin of any race. The terms White, Black, and Asian and Pacific Islanders (API), refer to non-Hispanic individuals self-classifying into these racial groups. The Raw Data panel reports the exact counts from Arias et al. (2016). The Adjusted for Race and Hispanic Origin Misclassification panel first adjust counts based on death certificate information for misclassification of race and Hispanic origin.*

## Methodological Supplement

### Introduction

The decomposition of demographic measures is a fundamental tool in a demographer’s toolkit. Most decomposition techniques focus on changes in demographic measures over time or, more generally, differences between demographic rates (Andreev, Shkolnikov, and Begun 2002; Arriaga 1984; Caswell 1989; Das Gupta 1978; Horiuchi, Wilmoth, and Pletcher 2008; Kitagawa 1964; Vaupel and Canudas-Romo 2002). Fewer techniques are available to researchers needing to estimate the contribution of different subpopulations to a demographic measure for the total population. Although some of the general decomposition techniques can be used for this purpose with some adjustments, they quickly become computationally inefficient even when few groups are involved. Below we develop a new decomposition technique which overcomes these limitations. See (Paglino 2024) for more details and replicable code.

### Development of the Decomposition Method

Given a population $(T)$ and a partition of the population into mutually exclusive subgroups $(G_{1},G_{2},\ldots,G_{N})$, how can we compute the contribution of subgroups $G_{1},G_{2},\ldots G_{N}$ to life table functions $(l_{x},_{n}L_{x}, ,_{n}T_{x}, e_{x}$) starting from the life table of a baseline population $(B)$? This paper proposes a decomposition method to answer this question and proves some of its properties.

The basic form of the decomposition method developed in this paper was originally proposed by Hendi and Ho (2021) to investigate the contribution of the foreign born population to the national life expectancy at age 1 in the United States (Hendi and Ho 2021). In that context, Hendi and Ho considered a total population $T$ including all residents of the United States, a baseline population $B$ including only US-born residents, and a single contributing group $G$ including all foreign-born residents. They proposed to compute the contribution of the foreign-born population to the national life expectancy at age 1 as:

$$C^{G}=e_{1}^{T}-e_{1}^{B}$$

Where $e_{1}^{T}$ is the life expectancy at age 1 for the total population and $e_{1}^{B}$ is the life expectancy at age 1 for US-born residents (the baseline population). The logic behind this formula is clear, the difference between the life expectancy of all US residents and US-born residents must be explained by the contribution of the foreign-born population.

### A Decomposition for $(\boldsymbol{N}=\boldsymbol{2})$

Let us consider a population $\left( T \right)$ that is composed of a baseline population $(B)$ and two mutually exclusive contributing groups ${(G}_{1},G_{2})$. Let us also denote the population combining ${(G}_{1},G_{2})$ as $G$. Finally, let us be as general as we can and leave the life table function of interest unspecified. As in other general decomposition techniques, we will see that computationally all that matters is that we can write the life table measure as a function of a single input vector storing exposures and deaths counts (Andreev et al. 2002; Caswell 1989; Horiuchi et al. 2008). We will denote with $l(A)$ the life table function for population $A$. To be precise, the generic $l(\cdot)$ function takes as input a vector of age-specific death counts and exposures for population $A$ (stacked to form a single vector) and outputs the life table measure $l(A)$. Hendi and Ho’s expression for the total contribution of the two groups is still valid:

$$C^{G}=l(T)-l(B)$$

Because both the exposures and the death counts involved in the computation of life tables combine additively, we have that $l(B)=l\left( T-G \right)$. Equivalently, we could write $l(T)=l(B+G)$. The two previous equations hold because once death counts and exposures for a population are stacked into a single vector, to obtain the corresponding vector for the combined population we just need to sum the two subpopulation-specific vectors. Together, these two equations allow us to write:

$$C^{G}=l\left( T \right)-l\left( B \right)=l\left( B+G \right)-l\left( B \right)=l\left( T \right)-l(T-G)$$

Looking at the equation above it is apparent that the total contribution of population $G$ can equivalently be understood as an addition process starting from the baseline population $C^{G}=l(B+G)-l(B)$ or as a subtraction process starting from the total population $C^{G}=l(T)-l(T-G)$. It now becomes useful to introduce another layer of notation. We define $C^{G+}\equiv l(B+G)-l(B)$ and call it the “addition contribution” of $G$. We instead define $C^{G-}\equiv l(T)-l(T-G)$ and call it the “subtraction contribution” of $G$. Clearly, when a single contributing population is considered $C^{G+}=C^{G-}$. However, we will see that even in the case of two contributing subpopulations $(G_{1},G_{2})$, $C^{1+}\neq C^{1-}$ and $C^{2+}\neq C^{2-}$ so that the notation I just introduced becomes useful. To see why these two inequalities arise, let us consider $C^{1+}$ and $C^{1-}$. Suppose for the moment that both $G_{1}$ and $G_{2}$ contribute positively to the life table function we are considering, and more precisely that $C^{1+},C^{1-},C^{2+},C^{2-}>0$. When we compute $C^{1-}$ we are then starting from a population $T$ which already encapsulates the positive effect of population $G_{2}$. On the contrary, when we compute $C^{1+}$ we are starting from the baseline population $B$ that has not yet received the positive effect of population $G_{2}$. Furthermore, population $B$ from which are starting in our calculations for $C^{1+}$ is smaller than population $B+G_{2}$ from which we start in our calculations for $C^{1-}$. Consequently, the effect of $G_{1}$ will be larger when computed as $C^{1+}$ than when computed as $C^{1-}$ and $C^{1+}>C^{1-}$. A specular argument can be made for $C^{2+},C^{2-}$. While the inequality $C^{1+}>C^{1-}$ depends on the assumption that both populations have a positive effect, it illustrates the general principle that will lead to the inequality $C^{1+}\neq C^{1-}$.

The inequality above means that we now have two different measures of the contribution of each group. One way of solving this inconsistency is to introduce a third type of contribution ${C^{1}\equiv\frac{1}{2}(C}^{1+}+C^{1-}$), which we will call the “average contribution”. It turns out that the average contributions have a nice property that both the addition and the subtraction contributions generally lack.

$$C^{1}+C^{2}={\frac{1}{2}(C}^{1+}+C^{1-})+ {\frac{1}{2}(C}^{2+}+C^{2-})$$

$$=\frac{1}{2}[\left( l(B+G_{1})-l(B) \right)+\left( l(T)-l(T+G_{1}) \right)+\left( l(B+G_{2})-l(B) \right)+\left( l(T)-l(T+G_{2}) \right)]$$

$$=\frac{1}{2}\left[ 2\left( l\left( T \right)-l\left( B \right) \right)+\left( l\left( B+G_{1} \right)-l\left( T-G_{2} \right) \right)+\left( l\left( B+G_{2} \right)-l\left( T-G_{1} \right) \right) \right]$$

$$=\left( l\left( T \right)-l\left( B \right) \right)+\frac{1}{2}\left[ \left( l\left( B+G_{1} \right)-l\left( T-G_{2} \right) \right)+\left( l\left( B+G_{2} \right)-l\left( T-G_{1} \right) \right) \right]$$

$$=C^{G}+\frac{1}{2}\left[ \left( l\left( B+G_{1} \right)-l\left( T-G_{2} \right) \right)+\left( l\left( B+G_{2} \right)-l\left( T-G_{1} \right) \right) \right]$$

$$=C^{G}+\frac{1}{2}\left[ \left( l\left( B+G_{1} \right)-l\left( B+G_{1} \right) \right)+\left( l\left( B+G_{2} \right)-l\left( B-G_{2} \right) \right) \right]$$

$$=C^{G}$$

So, the two average contributions sum to the total contribution of $G$, which establishes $C^{1},C^{2}$ as a legitimate decomposition of $C^{G}$. Note that the substitutions in the second last line hold because of the additivity of exposures and deaths so $l(T-G_{2})=l\left( B+G_{1}+G_{2}-G_{2} \right)=l\left( B+G_{1} \right)$ and similarly $l(T-G_{1})=l(B+G_{2})$. Notice also that no special properties of the life table function were used in the proof which establishes that this approach is valid for functions other than life expectancy as long as they can be written as a function of a vector of death counts and exposures.

### Extending the Decomposition to the Case $(\boldsymbol{N}=\boldsymbol{3})$

The case of three groups $(G_{1},G_{2},G_{3})$ introduces a few additional complications which illuminate some properties of this decomposition and will allow us to find a general expression for any number of groups. The main difference with the $\left( N=2 \right)$ case is that our notation $C^{n+},C^{n-}$ for $n=1,2,3$ is no longer sufficient to describe all possible ways of computing the contribution of group $G_{n}$ to the life table function for the total population. Indeed, we now have four ways of computing the contribution of $G_{1}$:

1. $l\left( B+G_{n} \right)-l(B)$
2. $l\left( B+G_{2}+G_{1} \right)-l(B+G_{2})$
3. $l\left( B+G_{3}+G_{1} \right)-l(B+G_{3})$
4. $l\left( T \right)-l(B+G_{2}+G_{3})$

Number 1 and 4 are equal to $C^{1+}$ and $C^{1-}$, but number 2 and 3 are outside of what we have seen so far. In the next section, I will introduce a more general notation to capture these cases. However, for the moment, let us focus on how to define the average contribution in this case. It turns out that the average contribution in this case assigns weights to each term that are inversely proportional to the number of groups $N=3$ and to the number of contributions involving the same number of subpopulations. Notice that contributions 1 and 4 involve 1 and 3 subpopulations respectively, while contributions 2 and 3 each involve 2 subpopulations. As such, contributions 1 and 4 have weight $\frac{1}{N}\frac{1}{1}=\frac{1}{3}$, while contributions 2 and 3 have weight $\frac{1}{N}\frac{1}{2}=\frac{1}{3}\frac{1}{\boldsymbol{2}}=\frac{1}{6}$. The use of these weights is equivalent to first averaging within contributions involving the same number of subpopulations and then averaging between contributions involving different numbers of subpopulations. While this might seem unintuitive, I will now show that it leads to a set of average contributions that sum to the total contribution as in the $(N=2)$ case.

$$C^{1}+C^{2}+C^{3}=\frac{1}{3}\left( l(B+G_{1})-l(B) \right)+ \frac{1}{6}\left( l\left( B+G_{2}+G_{1} \right)-l\left( B+G_{2} \right) \right)+ \frac{1}{6}\left( l\left( B+G_{3}+G_{1} \right)-l\left( B+G_{3} \right) \right)+ \frac{1}{3}\left( l\left( T \right)-l\left( B+G_{2}+G_{3} \right) \right)$$

$$+\frac{1}{3}\left( l(B+G_{2})-l(B) \right)+ \frac{1}{6}\left( l\left( B+G_{1}+G_{2} \right)-l\left( B+G_{1} \right) \right)+ \frac{1}{6}\left( l\left( B+G_{3}+G_{2} \right)-l\left( B+G_{3} \right) \right)+ \frac{1}{3}\left( l\left( T \right)-l\left( B+G_{1}+G_{3} \right) \right)$$

$$+\frac{1}{3}\left( l\left( B+G_{3} \right)-l\left( B \right) \right)+ \frac{1}{6}\left( l\left( B+G_{1}+G_{3} \right)-l\left( B+G_{1} \right) \right)+ \frac{1}{6}\left( l\left( B+G_{2}+G_{3} \right)-l\left( B+G_{2} \right) \right)+ \frac{1}{3}\left( l\left( T \right)-l\left( B+G_{1}+G_{2} \right) \right)$$

It is easy to see that one can take out of this complicated expression the target value $l(T)-l(B)$. We can then reorder the remaining terms and write:

$$C^{1}+C^{2}+C^{3}=\left( l\left( T \right)-l\left( B \right) \right)+ \frac{1}{3}\left( l(B+G_{1})+l\left( B+G_{2} \right)+l(B+G_{3}) \right)$$

$$- \frac{1}{3}\left( l\left( B+G_{1}+G_{2} \right)+l\left( B+G_{1}+G_{3} \right)+l\left( B+G_{2}+G_{3} \right) \right)$$

$$- \frac{1}{6}\left( 2l(B+G_{1})+ 2l(B+G_{2})+2l(B+G_{3}) \right)$$

$$+ \frac{1}{6}(2l\left( B+G_{1}+G_{2} \right)+2l\left( B+G_{1}+G_{3} \right)+2l\left( B+G_{2}+G_{3} \right))$$

From which one can easily verify that $C^{1}+C^{2}+C^{3}=\left( l\left( T \right)-l\left( B \right) \right)$ as we wanted to prove. This result establishes $\left( C^{1},C^{2},C^{3} \right)$ as a legitimate decomposition of $C^{G}$.

By comparing the computations involved in the case $\left( N=2 \right)$ to those for the case $\left( N=3 \right)$, it becomes apparent that the computational requirement for this method increases very fast as the number of groups increases. However, the number of groups in the typical demographic application will not be too large and I will show that compared to alternative approached, this decomposition method is computationally very fast.

### Generalizing the Decomposition to a Population with $\boldsymbol{N}$ subgroups

To study the general case of $N$ contributing groups, it is useful to introduce a slightly more general notation. We define:

$$C^{n,m,i}=l(B+G_{i}^{m}+G_{n})-l(B+G_{i}^{m})$$

With $n=\left\{ 1,2,\ldots,N \right\}, m=\left\{ 0,1,\ldots,N-1 \right\}, i=\{1,2,\ldots,\binom{N-1}{m}\}$ and where $G_{i}^{m}$ is the $i^{th}$ set of subpopulations $G_{k}$ with $k\neq n$ such that $\#G_{i}^{m}=m$. In words, $C^{n,m,i}$ is the contribution of subgroup $G_{n}$ to the life table function $l(\cdot)$ for the total population calculated as the difference between the value of $l(\cdot)$ for the population obtained by combining the baseline population, the $i^{th}$ possible subset of $G_{k}$’s not including $G_{n}$, and $G_{n}$ ($l(B+G_{i}^{m}+G_{n})$), and the value of $l(\cdot)$ for the population obtained by combining the baseline population and the $i^{th}$ possible subset of $G_{k}$’s not including $G_{n}$ ($l(B+G_{i}^{m})$). This definition might seem confusing but it’s just a generalization of the types of contributions we have seen in the cases $N=2, 3$. Indeed, for $N=2$, our familiar $C^{1+}$ is simply $C^{1,0,1}$ while $C^{1-}$ is $C^{1,1,1}$. For $N=3$, we have the more interesting “internal” terms for which we had no notation so far. With this new notation we can write:

1. $l(B+G_{2}+G_{1})-l(B+G_{2})=C^{1,1,1}$
2. $l(B+G_{3}+G_{1})-l(B+G_{3})=C^{1,1,2}$

With these expressions, we can see why $m$ should be bounded between $0$ and $N-1$ and $i$ between $1$ and $\binom{N-1}{m}$. With $m=0,$ we recover $C^{1+}$, when $m=0$, there is no need for an index $i$ which we can conventionally set to $1$. For $m=1$, $G_{i}^{m}$ contains only one element which we can choose out of the $N-1$ groups that are not $G_{n}$. The first of these groups will be denoted as $G_{1}^{1}$, the second as $G_{2}^{1}$, and so on. The order in which we select the groups does not matter because addition is commutative. Notice that, for a general value $m$ there will be $\binom{N-1}{m}$ of these groups, which explains the limits imposed on $i$. Finally, when we reach $m=N-1$, we obtain $C^{1-}$ because:

$$l\left( B+G_{1}^{N-1}+G_{1} \right)-l(B+G_{1}^{N-1})=l(T)-l(T-G_{1})=C^{1-}$$

Clearly, there is only one way of choosing $N-1$ elements from a set of $N-1$ elements.

As for the $N=3$ case, we now need to find appropriate weights for each contribution $C^{n,m,i}$. The general principle is still the same, we need weights that are inversely proportional to the number of contribution types $N$ and inversely proportional to the number of contributions for the specific type $m$. This consideration leads to a simple expression:

$$w^{m}=\frac{1}{N\binom{N-1}{m}}$$

Which is just a generalization of the weights we derived for $(N=3)$. In the expression above, $w^{m}$ denotes the weight for all terms $C^{n,m,i}$. These weights are equal for all $m$-type contributions and ensure that each set of $m$-contributions is collectively assigned the same weight $\frac{1}{N}$ while each of its members is also assigned the same weight $\frac{1}{N\binom{N-1}{m}}$. With our new notation and having defined appropriate weights, we can define the average contribution of group $G_{n}$ as:

$$C^{n}=\sum_{m=0}^{N-1} \frac{1}{N}\sum_{i=1}^{\binom{N-1}{m}} \frac{1}{\binom{N-1}{m}}C^{n,m,i}$$

To establish that average contributions defined in this way form a valid decomposition of $C^{G}$ into group-specific contributions, we just need to prove that:

$$\sum_{n=1}^{N} C^{n}=l(T)-l(B)$$

We already saw that for $N=2, 3$, the proof involves showing that all terms except $l(T)$ and $l(B)$ have weights summing to $0.$ This is harder to do directly now that we have many terms. However, we can start by recognizing that the life table function computed for a given population composed by a set of subgroups plus the baseline population can appear in two ways:

1. As a term of the form $l(B+G_{i}^{m}+G_{n})$ in a contribution of the type $C^{n,m,i}$
2. As a term of the form $l\left( B+G_{i}^{m+1} \right)$ in a contribution of the type $C^{k,m,i}$ where $k\neq n$.

In the first case, the life table function will have weight $w^{m}$ and in the second case it will have weight $-w^{m+1}$. To understand what the final weight will be for each term $l(B+G_{i}^{m}+G_{n})$, we just need to know how many times it will appear with weight $w^{m}$ and how many times with weight $w^{m+1}$. For terms of the first type, we have $m+1$ ways of choosing $n$ while keeping the subgroups involved the same. On the other hand, terms of the second type can only appear in contributions involving the $N-\left( m+1 \right)$ excluded groups. With this information, we are now able to compute the weight associated with the life table function computed for each population:

$$w\left( l({B+G}_{i}^{m}+G_{n}) \right)= \left( m+1 \right)w^{m}-\left( N-m-1 \right)w^{m+1}$$

$$=\left( m+1 \right)\frac{1}{N\binom{N-1}{m}}-\left( N-m-1 \right)\frac{1}{N\binom{N-1}{m+1}}$$

$$=\left( m+1 \right)\frac{\left( N-1 \right)!}{m!\left( N-1-m \right)!}-\left( N-m-1 \right)\frac{\left( N-1 \right)!}{\left( m+1 \right)!\left( N-1-m-1 \right)!}$$

$$=\left( m+1 \right)\frac{m!\left( N-1-m \right)!}{\left( N-1 \right)!}-\left( N-m-1 \right)\frac{\left( m+1 \right)!\left( N-1-m-1 \right)!}{\left( N-1 \right)!}$$

$$=\frac{(m+1)!\left( N-1-m \right)!}{\left( N-1 \right)!}-\frac{\left( m+1 \right)!\left( N-1-m \right)!}{\left( N-1 \right)!}$$

$$=0$$

Which proves that life table functions for all populations of the type ${B+G}_{i}^{m}+G_{n}$cancel out. The only two exceptions are $l(B)$, which appears $N$ times with weight $-\frac{1}{N}$, and $l(T)$, which appears $N$ times with weight $\frac{1}{N}$. Thus, summing the average contributions for our $N$ groups we obtain:

$$\sum_{n=1}^{N} C^{n}=l(T)-l(B)$$

as desired. This result shows that the average contributions provide a decomposition of the total contribution $C^{G}$ even in the case of $N$ subgroups.

## References

Andreev, Evgueni M., Vladimir M. Shkolnikov, and Alexander Z. Begun. 2002. “Algorithm for Decomposition of Differences between Aggregate Demographic Measures and Its Application to Life Expectancies, Healthy Life Expectancies, Parity-Progression Ratios and Total Fertility Rates.” *Demographic Research* 7:499–522.

Arias, Elizabeth, Melonie Heron, National Center for Health Statistics, Jahn Hakes, and US Census Bureau. 2016. “The Validity of Race and Hispanic-Origin Reporting on Death Certificates in the United States: An Update.” *Vital and Health Statistics. Series 2, Data Evaluation and Methods Research* (172):1–21.

Arias, Elizabeth, Carolyn Liebler, Marc A. Garcia, and Rogelio Sáenz. 2025. “Data Impacts of Changes in U.S. Census Bureau Procedures for Race and Ethnicity Data.” *SSM - Population Health* 101742. doi: 10.1016/j.ssmph.2024.101742.

Arias, Elizabeth, and Jiaquan Xu. 2022a. “United States Life Tables, 2019.” *National Vital Statistics Reports: From the Centers for Disease Control and Prevention, National Center for Health Statistics, National Vital Statistics System* 70(19):1–59.

Arias, Elizabeth, and Jiaquan Xu. 2022b. *United States Life Tables, 2020*. National Center for Health Statistics (U.S.). doi: 10.15620/cdc:118055.

Arriaga, Eduardo E. 1984. “Measuring and Explaining the Change in Life Expectancies.” *Demography* 21(1):83–96. doi: 10.2307/2061029.

Camarda, Carlo G. 2012. “MortalitySmooth: An R Package for Smoothing Poisson Counts with P-Splines.” *Journal of Statistical Software* 50:1–24. doi: 10.18637/jss.v050.i01.

Caswell, Hal. 1989. “Analysis of Life Table Response Experiments I. Decomposition of Effects on Population Growth Rate.” *Ecological Modelling* 46(3):221–37. doi: 10.1016/0304-3800(89)90019-7.

Das Gupta, Prithwis. 1978. “A General Method of Decomposing a Difference between Two Rates into Several Components.” *Demography* 15(1):99–112. doi: 10.2307/2060493.

Gompertz, Benjamin. 1825. “On the Nature of the Function Expressive of the Law of Human Mortality, and on a New Mode of Determining the Value of Life Contingencies.” *Philosophical Transactions of the Royal Society of London* 115:513–83.

Greville, T. N. E. 1943. “Short Methods of Constructing Life Tables.” *Record from the American Institute of Actuaries* 32:29–42.

Hendi, Arun S., and Jessica Y. Ho. 2021. “Immigration and Improvements in American Life Expectancy.” *SSM - Population Health* 15:100914. doi: 10.1016/j.ssmph.2021.100914.

Horiuchi, Shiro, John R. Wilmoth, and Scott D. Pletcher. 2008. “A Decomposition Method Based on a Model of Continuous Change.” *Demography* 45(4):785–801. doi: 10.1353/dem.0.0033.

IPUMS. 2025. “Replicate Weights in the American Community Survey.” Retrieved May 5, 2025 (https://usa.ipums.org/usa/repwt.shtml).

Jones, Nicholas, Rachel Marks, Roberto Ramirez, and Merarys Ríos-Vargas. 2021. “Improved Race, Ethnicity Measures Show U.S. Is More Multiracial.” Retrieved April 2, 2024 (https://www.census.gov/library/stories/2021/08/improved-race-ethnicity-measures-reveal-united-states-population-much-more-multiracial.html).

Keyfitz, Nathan. 1966. “A Life Table That Agrees with the Data.” *Journal of the American Statistical Association* 61(314):305–12. doi: 10.1080/01621459.1966.10480866.

Keyfitz, Nathan, and Hal Caswell. 2005. *Applied Mathematical Demography*. New York: Springer-Verlag.

Keyfitz, Nathan, and James Frauenthal. 1975. “An Improved Life Table Method.” *Biometrics* 31(4):889–99. doi: 10.2307/2529814.

Kitagawa, Evelyn M. 1964. “Standardized Comparisons in Population Research.” *Demography* 1(1):296–315. doi: 10.1007/BF03208469.

Manson, Steven, Jonathan Schroeder, David Van Riper, Katherine Knowles, Tracy Kugler, Finn Roberts, and Steven Ruggles. 2023. “IPUMS National Historical Geographic Information System: Version 18.0 [Dataset]. Minneapolis, MN: IPUMS. 2023.”

Paglino, Eugenio. 2024. “The Groupwise Decomposition: Estimating Group-Specific Contributions to Life Table Functions.” doi: 10.5281/zenodo.11479754.

Preston, Samuel H., Patrick Heuveline, and Michel Guillot. 2001. *Demography : Measuring and Modeling Population Processes*. Oxford ; Blackwell Publishers,.

Riffe, Tim, Jose Manuel Aburto, Monica Alexander, Sean Fennell, Sarah Hertog, and Marius D. Pascariu. 2019. “DemoTools: An R Package of Tools for Aggregate Demographic Analysis.”

Rizzi, Silvia, Jutta Gampe, and Paul H. C. Eilers. 2015. “Efficient Estimation of Smooth Distributions From Coarsely Grouped Data.” *American Journal of Epidemiology* 182(2):138–47. doi: 10.1093/aje/kwv020.

Ruggles, Steven, Sarah Flood, Ronald Goeken, Megan Schouweiler, and Matthew Sobek. 2022. “IPUMS USA: Version 12.0, American Community Survey, 1-Year Samples 2017-2019.”

US Census Bureau. 2020. “Annual Estimates of the Resident Population by Sex, Age, Race, and Hispanic Origin for the United States: April 1, 2010 to July 1, 2019 (NC-EST2019-ASR6H).”

US Census Bureau. 2023. “Annual Estimates of the Resident Population by Sex, Age, Race, and Hispanic Origin for the United States: April 1, 2020 to July 1, 2022 (NC-EST2022-ASR6H).”

Vaupel, James W., and Vladimir Canudas-Romo. 2002. “Decomposing Demographic Change into Direct vs. Compositional Components.” *Demographic Research* 7:1–14.
